# Accurate TCR-pMHC interaction prediction using a BERT-based transfer learning method

**DOI:** 10.1093/bib/bbad436

**Published:** 2023-12-01

**Authors:** Jiawei Zhang, Wang Ma, Hui Yao

**Affiliations:** Fresh Wind Biotechnologies Inc. (Tianjin), Tianjin, China; Fresh Wind Biotechnologies Inc. (Tianjin), Tianjin, China; Fresh Wind Biotechnologies USA Inc., Houston, TX, USA

**Keywords:** prediction of TCR-pMHC interaction, immunotherapy, deep learning, representation learning, BERT

## Abstract

Accurate prediction of TCR-pMHC binding is important for the development of cancer immunotherapies, especially TCR-based agents. Existing algorithms often experience diminished performance when dealing with unseen epitopes, primarily due to the complexity in TCR-pMHC recognition patterns and the scarcity of available data for training. We have developed a novel deep learning model, ‘TCR Antigen Binding Recognition’ based on BERT, named as TABR-BERT. Leveraging BERT's potent representation learning capabilities, TABR-BERT effectively captures essential information regarding TCR-pMHC interactions from TCR sequences, antigen epitope sequences and epitope-MHC binding. By transferring this knowledge to predict TCR-pMHC recognition, TABR-BERT demonstrated better results in benchmark tests than existing methods, particularly for unseen epitopes.

## INTRODUCTION

The recognition of antigen epitopes presented by major histocompatibility complex (MHC) molecules by T cell receptor (TCR) binding is the crucial initial step in adaptive immunity [1], also key to various cancer immunotherapies such as checkpoint inhibitors [2], cancer vaccines [3] and TCR-based agents [4, 5]. The specificity of TCR lies predominantly in the variable regions (Vα, Vβ), with the most variable region known as complementarity determining region 3 (CDR3), determining the antigen binding [6].TCR-antigen-MHC interactions, commonly referred to as TCR-pMHC interactions, display polymorphic recognition patterns due to the extensive diversity in amino acid sequences of TCRs and MHC molecules among individuals, posing significant challenges for an accurate identification [7].

Identification of TCR-pMHC interactions conventionally involves antigen stimulation of T cells, followed by laborious in-vitro sorting and amplification of epitope-specific T cells [8]. Recent advancements in next-generation sequencing technologies, particularly the combination of single-cell RNA sequencing (scRNA-Seq) and TCR sequencing (scTCR-seq), offer an effective tool to characterize TCR diversity and clonality in response to specific antigens [9]. Nevertheless, despite these advances, the experimental methods for characterizing TCR-pMHC interactions remain time-consuming and expensive, hindering the rapid progress of immunotherapies [10].

The emergence of computational prediction methods has significantly contributed to the accurate and rapid identification of TCR-pMHC interactions. These methods can be broadly classified into two categories: epitope-specific models and pan-epitope models. Epitope-specific models utilize separate prediction models for specific epitopes, which restricts their application potential. In contrast, pan-epitope models enable the prediction of binding for unseen epitopes with specific TCRs using diverse deep learning approaches.

Numerous pan-epitope models have been developed and are currently available. Examples of such models include DLpTCR [11], which is an ensemble model integrating pTCRα and pTCRβ information. ERGO-II [12] leverages multiple input sources like epitopes, MHC subtypes, T cell type, CDR3α and CDR3β chains and corresponding V and J genes. ImRex [13] and TEIM [14] utilize convolutional neural networks (CNNs) [15] to capture physicochemical properties and contact information of TCR and epitope sequences, respectively. Moreover, PanPep [16] employs neural turing machines (NTMs) to enhance model robustness, particularly when confronted with unseen epitopes. Lastly, pMTnet [17] adopts a transfer learning model, leveraging pre-trained models to transfer TCR sequence information and pMHC binding knowledge to improve TCR-pMHC prediction performance.

Despite significant advancements, several limitations persist in current computational methods for TCR-pMHC interaction prediction. Most methods, including DLpTCR, ImRex, PanPep and TEIM, do not effectively utilize the abundant unlabeled TCR sequence data. Additionally, except for ERGO-II and pMTnet, the models do not consider the MHC subtype information. Furthermore, except for pMTnet, the crucial pMHC binding information is largely disregarded. The embedding methods employed by pMTnet tend to memorize training data, leading to poor generalization for predicting unseen epitopes [16]. Effectively leveraging the existing ‘big data’ from unlabeled TCR sequences, epitope sequences, MHC subtypes and pMHC binding information remains a challenging issue to further enhance the prediction of TCR-pMHC interactions.

To tackle these challenges, we present a TCR-Antigen Binding Recognition model based on Bidirectional Encoder Representation from Transformer (TABR-BERT). This is a transfer learning model with three sub-models: (1) a TCR embedding model (TCR-BERT), (2) a pMHC embedding model (pMHC-BERT) and (3) a multilayer perceptron (MLP)-based TCR-pMHC prediction model. TABR-BERT leverages BERT [18] to effectively learn from vast unlabeled TCR sequences and pMHC complex data. With BERT's contextual understanding capabilities, our model adeptly discerns and encodes pivotal residues involved in TCR-pMHC interactions. Our model significantly improved prediction performance on benchmark tests, particularly for predicting binding with unseen epitopes.

## METHODS

TABR-BERT is divided into three parts: TCR embedding model, pMHC embedding model and TCR-pMHC prediction model.

### TCR embedding model (TCR-BERT)

#### Architecture

**Figure 1:**
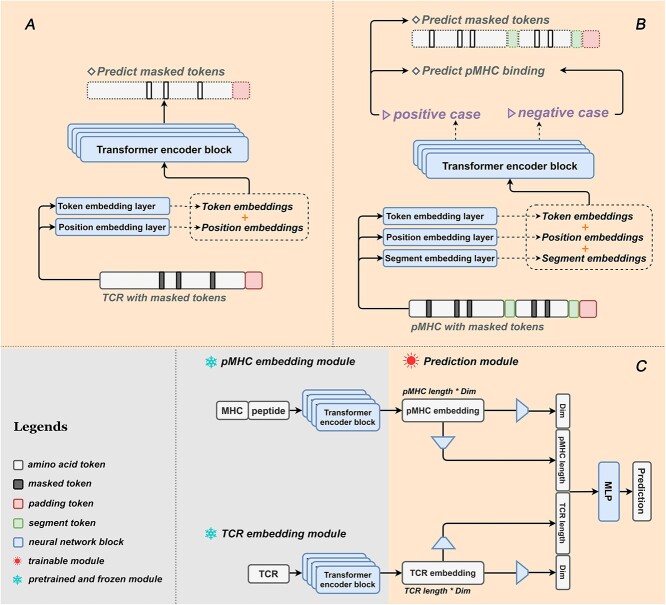
The architecture of TABR-BERT. The architectures of the TCR-BERT, the pMHC-BERT and the TCR-pMHC prediction model are illustrated in panels **A**, **B** and **C**, respectively. The solid rounded rectangle represent the neural networks with trainable parameters. The trapezoids in C indicate the mapping layers that reduce the dimensions of embedding matrices. "TCR length" and "pMHC length" denote the lengths of TCR sequences, epitope sequences and MHC pseudo-sequences. "Dim" represents the dimensions of the embedding vectors for each amino acid. A "token" represents an atomic unit within the embedding. Specifically, the amino acid token corresponds to individual amino acids. The masked token serves to conceal amino acids during training. The padding token serves to standardize input sequences to a uniform length and enables the model to discern the true length of the input amino acid sequence. The segment token is employed to separate between MHC pseudo-sequences, epitope sequences and padding sequences. During training the TCR-pMHC prediction model in C, the parameters of the TCR-BERT and pMHC-BERT models in A and B, respectively, are frozen.

TCR-BERT is a pre-trained architecture (Figure 1A) based on BERT, comprising four transformer encoder layers [19] with 256 embedding dimensions and 8 attention heads at each layer. It also incorporates a position embedding layer and a token embedding layer, both with learnable parameters.

#### Model training

For training the TCR-BERT, CDR3β sequences from the TCR β chain were utilized as representatives for the entire TCR due to their high diversity in TCR-pMHC interaction. A training dataset, Tr-TCR, of 113 529 384 unique TCR CDR3β sequences with lengths from 10 to 30 residues from TCRdb [20] was gathered, covering over 29 diseases and 10 tissues.

The model was trained using the masked language modeling (MLM) task [18] with cross-entropy loss [21]. The AdamW [22] optimizer with a learning rate of 0.0005 and a batch size of 512 was used. A learning rate warm-up strategy [23] was applied during the initial 4000 training steps, and a learning rate decay strategy [23] was implemented, reducing the learning rate by 0.3 if the validation loss did not decrease for 2 consecutive epochs. Training continued for a maximum of 100 epochs, with early stopping if the validation loss did not decrease for 4 consecutive epochs.

### pMHC embedding model (pMHC-BERT)

#### Architecture

pMHC-BERT (Figure 1B) shares a similar architecture to the TCR embedding model, consisting of 4 transformer encoder layers with 256 embedding dimensions and 8 attention heads at each layer. It also includes learnable position, token and segment embedding layers. The segment embedding layer facilitated amino acid differentiation between the MHC-I molecules and the epitope by learning two distinct vectors, one specific to MHC-I and the other to the epitope. This mechanism enables the model to capture unique features of amino acids between MHC-I and epitope sequences, effectively decoupling their representations.

#### Model training

The training data were downloaded from seven datasets on IEDB [24–30], resulting in 4 130 113 unique data entries. After removing the potential data leakage whose epitopes exist in TABR-BERT benchmark datasets, 4 128 332 data entries, including 179 801 binding affinity (BA) and 3 948 531 mass spectrometry (MS) data with 3 788 848 specific epitopes and 175 MHC-I molecules, form the final training dataset, Tr-pMHC. Among them, the BA data were transformed to a 0–1 scale using the method in MHCflurry2.0 [31].

pMHC-BERT was trained using two loss functions: cross-entropy loss for the selective masked language modeling (SMLM) task and mean square error (MSE) loss for the next sentence prediction (NSP) task [18]. They were combined into an overall loss function for training the model. The SMLM task was employed to predict masked amino acids selectively in the cases where the input epitope and MHC binding occurs. Its inclusion aimed to mitigate interference and avoid spurious information acquisition during the training process. The training strategy and hyperparameters remained consistent with those used for the TCR embedding model. (See details of TCR-BERT and pMHC-BERT training in Supplementary Methods 1 and 2 available online at http://bib.oxfordjournals.org/, respectively.)

### TCR-pMHC prediction model

#### Architecture

The TCR-pMHC prediction model (Figure 1C) consists of four mapping layers and an MLP [23] layer. The mapping layers flatten TCR or pMHC embedding matrices to two one-dimension vectors per row and column, respectively. This process, reminiscent of Low-Rank Adaptation (LoRA) [32], is commonly employed in transfer learning to distill key information and simplify downstream modeling. The prediction MLP layer includes dense layers with 200, 100 and 50 neurons activated by RELU, followed by dropout layers with a rate of 0.4. The final layer has a single neuron with tanh activation.

#### Model training

The TCR-pMHC prediction model was trained and tested using data from four publicly available datasets: IEDB [30], McPAS [33], VDJdb [34] and PIRD [35]. After preprocessing, 71 836 TCR-pMHC positive pairs were obtained, containing 64 967 unique CDR3β sequences, 624 unique epitopes and 64 unique MHCs, forming the master TCR-pMHC set (see the construction of the dataset in Supplementary Method 3.1 available online at http://bib.oxfordjournals.org/).

The master set was split into a training set (Tr-TCR-pMHC) and an independent benchmark test set (Te-S1, see details in Benchmark Testing). Tr-TCR-pMHC comprised 70 423 positive examples with 127 unique epitopes, ensuring the inclusion of epitopes with >10 occurrences in the master set. Additionally, we removed cases with their epitopes involved in any zero-shot benchmark tests. Negative cases were generated by randomly matching pMHC in positive TCR-pMHC pairs with TCR sequences from a healthy TCR dataset collected from 587 healthy volunteers' peripheral blood [36, 37].

For model training, we employed a contrast learning methodology [17] for the TCR-pMHC prediction model. This involved simultaneously feeding positive and negative data pairs into two identical models with shared weights. The contrast learning loss function, defined as:


$$ \mathrm{loss}=\mathrm{Relu}\left(1+{f}^{-}-{f}^{+}\right)+0.2\ast \left({f^{-}}^2+{f^{+}}^2\right) $$

was utilized to optimize the distances between positive and negative cases in the searching space. Here, ${f}^{-}$ and ${f}^{+}$ represented model outputs of negative and positive cases, respectively.

The TCR-pMHC prediction model was optimized using an AdamW optimizer with a learning rate of 0.005, a batch size of 256 and a learning rate decay strategy that is same as the pre-training models. The maximum epoch was set to 100, and training stopped if the validation loss did not decrease for 6 consecutive epochs. The final percentile rank score was estimated from the ensemble distribution consisting of the cases randomly matching positive pMHC complexes with 1000 TCRs from the healthy populations. (See the details of the TCR-pMHC prediction model training and prediction in Supplementary Methods 3.2–3.4 available online at http://bib.oxfordjournals.org/.)

### Benchmark testing

We conducted evaluations on four distinct test sets to benchmark six TCR-pMHC binding prediction models: DLpTCR, ERGO-II, ImRex, PanPep, pMTnet and TEIM. (See details of benchmark models in Supplementary Method 5 available online at http://bib.oxfordjournals.org/.) All test sets are independent from the training sets. The testing process comprises two settings: non-zero-shot and zero-shot. In the non-zero-shot setting, the test sets include epitopes that appear (are seen) in the training set. Conversely, in the zero-shot setting, all epitopes are unseen during training, evaluating the models’ generalization capability.

The test sets consist of experimentally determined positive examples and an equal number of negative examples that randomly match pMHCs with TCR from a healthy population. Specifically, we utilized four test sets: Te-S1, Te-S2, Te-S3 and Te-S4. Te-S1, a subset of the master TCR-pMHC set, contains 1413 positive cases with 497 unique epitopes, and it is non-zero-shot for all models except TABR-BERT, which operates under the zero-shot setting. Te-S1 is the largest testing set and presents a more stringent evaluation task for TABR-BERT than other models.

Te-S2 includes 618 positive examples from the pMTnet test set [17], with 222 unique epitopes, and is non-zero-shot for all models. Te-S3 comprises 397 positive examples from the PanPep test set [16], encompassing 266 unique epitopes, and is zero-shot for all models.

**Figure 2:**
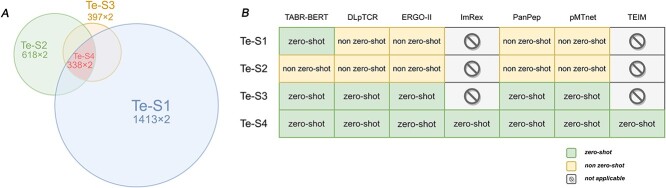
Illustration of benchmark test sets. Panel **A** depicts the relationship between all benchmark sets. The "×2" indicates that the datasets comprise experimentally determined positive cases matched with an equal number of randomly generated negative cases. Panel **B** shows the status of being zero-shot and non-zero-shot for each benchmark model per test set.

Lastly, to thoroughly evaluate all seven models, including TEIM and ImRex models that require highly restrictive TCR and pMHC inputs, in the zero-shot setting, we constructed the Te-S4 test set. By combining positive cases from Te-S1, Te-S2 and Te-S3 while removing duplicates and TCR-pMHC pairs that TEIM and ImRex cannot handle, we generated a test set containing 338 TCR-pMHC positive examples with 183 unique epitopes. Consequently, Te-S4 served as a zero-shot evaluation for all seven models. The relationship among all four datasets and their non-zero/zero-shot status for each model are illustrated in Figure 2.

We evaluated model performance using two key metrics: the area under the receiver operating characteristic curve (AUC-ROC) and the area under the precision-recall curve (AUC-PR). AUC-ROC measures the classifier’s ability to distinguish between positive and negative samples, where a value of 0.5 suggests random classification. On the other hand, AUC-PR assesses the model’s capacity to maintain both high precision and high recall during classification, indicating better performance when the AUC-PR value is higher.

### Methods for deciphering the TCR and pMHC embedding model

We employed various statistical and quantitative measurements to assess the functional and structural importance of residues in TCR and epitope sequences. Firstly, we computed attention scores to quantify residue importance within the embedding models for TCR and epitope sequences, respectively. Subsequently, the GLIPH2 algorithm was utilized to identify sequence motifs associated with significant biological functions in TCR sequences. Finally, we calculated the average distances between TCR and epitope residues when interaction structures were available. (See detailed calculations in Supplementary Methods 4.1–4.4 available online at http://bib.oxfordjournals.org/.)

To assess the ability of our pMHC embedding model to capture epitope-MHC-I binding information, we utilized it to predict binding between epitopes and MHC class I molecules. Initially, we assembled an independent multi-allele test set (Te-pMHC) consisting of 9 158 100 epitope-MHC-I pairs sourced from 76 individuals with a true positive rate of 1%. Te-pMHC was curated from a compilation of 10 studies that employed mass spectrometry (MS) to identify MHC class I-bound peptides. It encompasses 71 distinct MHC types and comprises a total of 7 121 288 unique epitopes [31]. (For further elaboration, please refer to Supplementary Table 1 available online at http://bib.oxfordjournals.org/.) Subsequently, we connected the pMHC-BERT embedding to a basic MLP layer for prediction, without the need for additional training, as this model had already been employed in the training of pMHC embeddings for the NSP task. (Refer to Supplementary Method 4.5, available online at http://bib.oxfordjournals.org/, for a detailed description.)

### Construction of a dataset of TCRs targeting hotspot TP53 mutations

We initially curated eight hotspot TP53 missense mutations that are MHC-1 restricted, representing 3.717% of TP53 mutation-bearing cancer patients [38]. (See details in Supplementary Table 2 available online at http://bib.oxfordjournals.org/.) Neoantigen-reactive TCRs were identified through immunologic screening, and their CDR3β sequences were determined via deep sequencing [38]. Subsequently, we obtained mutant epitope sequences for each hotspot mutation using netMHCpan4.1 [28], as these sequences were not available in the original publication. Specifically, we ranked epitopes with lengths of 8–11 spanning the missense mutations, removed the ones overlapped with our training set and selected the top 5 hits as the epitopes for positive TCR-pMHC pairs. Finally, for each positive case, we paired it with 100 randomly selected negative cases from a healthy T cell receptor (TCR) dataset as described in Supplementary Method 3.2, resulting in a final test set comprising 80 positive and 8000 negative cases, thereby maintaining a 1:100 ratio, as outlined in Supplementary Table 5 available online at http://bib.oxfordjournals.org/. To evaluate prediction robustness, we generated 100 distinct test sets of same size by randomly selecting negative samples.

## RESULTS

We introduce TABR-BERT, a novel approach for predicting TCR-pMHC interaction, especially in scenarios involving previously unseen epitopes. Our methodology leverages transfer learning techniques based on the BERT model structure to effectively incorporate TCR sequence information, pMHC sequence information and binding capability data into representative embedding matrices. This approach mitigates the challenge of limited training data in TCR-pMHC binding prediction models. TABR-BERT demonstrated consistently superior performance compared to existing models across four independent test sets. These findings underscored the enhanced predictive accuracy and generalization capability of TABR-BERT approach.

###  

#### TABR-BERT exhibited superior predictive capabilities over state-of-the-art models in both zero-shot and non-zero-shot tests

**Figure 3:**
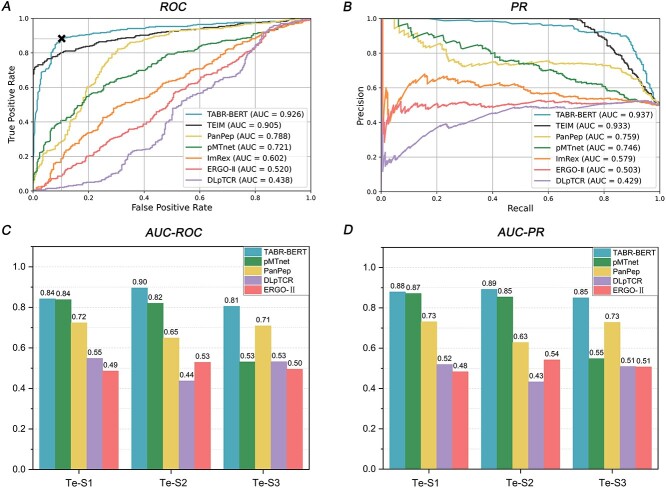
TABR-BERT outperforms state-of-the-arts models in all benchmark tests. Panel **A** showcases the receiver operating characteristic (ROC) curves of DLpTCR, ERGO-II, ImRex, PanPep, pMTnet, TEIM and TABR-BERT for Te-S4. ‘X’ indicates the optimal cutoff point, calculated at a prediction rank score of 0.901 by the Youden index for TABR-BERT. Panel **B** illustrates the precision-recall (PR) curves of these models for Te-S4. Panel **C** displays the AUC-ROC values for TABR-BERT, pMTnet, PanPep, DLpTCR and ERGO-II for Te-S1, Te-S2 and Te-S3. Panel **D** shows the AUC-PR values for these models for Te-S1, Te-S2 and Te-S3. The models are color-coded consistently across the panels, and AUC stands for the area under the curve.

In the zero-shot setting (Te-S4), TABR-BERT outperformed all other models, including recent deep learning approaches, with an AUC-ROC of 0.926 and an AUC-PR of 0.937 (Figure 3A and B). The closest competitor, TEIM, achieved an AUC-ROC of 0.905 and an AUC-PR of 0.933, but its limited applicability to various sequences due to stringent input requirements leads to its exclusion from other tests. PanPep and pMTnet ranked 3rd and 4th, respectively, with AUC-ROC values of 0.788 and 0.721, and AUC-PR values of 0.759 and 0.746. In contrast, ERGO-II and DLpTCR performed poorly, approaching random guess levels. The generalization capability of TABR-BERT for predicting unseen epitopes was further confirmed on a larger test set, Te-S3 (Figure 3C and D), where its AUC-ROC and AUC-PR performance significantly outperforms other models.

In the non-zero-shot scenario, TABR-BERT demonstrated the most robust performance in Te-S2 (Figure 3C and D), achieving the highest AUC-ROC of 0.896 and AUC-PR of 0.892. pMTnet ranks second with an AUC-ROC of 0.820 and AUC-PR of 0.854. However, pMTnet’s predictive performance significantly declined when handling unseen epitopes, achieving an AUC-ROC of 0.531 and AUC-PR of 0.548 in Te-S3. Lastly, in the largest test set, Te-S1, which presented a more stringent test for TABR-BERT as it includes seen epitopes for all models except TABR-BERT (Figure 2B), our model continued to outperform others with an AUC-ROC of 0.842 and AUC-PR of 0.879. These results strongly reinforced TABR-BERT’s excellent prediction capability for TCR-pMHC interactions across all tested scenarios.

The performance of TABR-BERT remained stable across various hyperparameter selections, training data sizes and the presence of high-frequency epitopes in the training set. We also investigated epitope sequence similarities between training and test sets for correct and incorrect predictions. Correct predictions had statistically significantly lower epitope sequence similarities compared to incorrect ones in Te-S1 (median of 1.60 versus 1.62, *P*-value = 0.026 by Wilcoxon rank-sum test), while no significant difference was observed in Te-S2, Te-S3 and Te-S4.

Lastly, we evaluated the model's performance on unseen TCRs by excluding 250 TCR sequences overlapping with the test set. The results showed robust performance, albeit with a minor decrease in AUC-ROC and AUC-PR from 0.842 and 0.879 to 0.829 and 0.871, respectively, for Te-S1. For detailed analyses and results on hyperparameter influence, training set size, high-frequency epitopes and sequence similarity, refer to Supplementary Analyses 6.1, 6.3, 6.4 and 6.5 available online at http://bib.oxfordjournals.org/. Additional performance breakdown based on TCR, epitope and MHC-I molecule distributions can be found in Supplementary Analysis 6.2 available online at http://bib.oxfordjournals.org/, and analysis of performance on unseen TCRs is available in Supplementary Analysis 6.6 available online at http://bib.oxfordjournals.org/.

**Figure 4:**
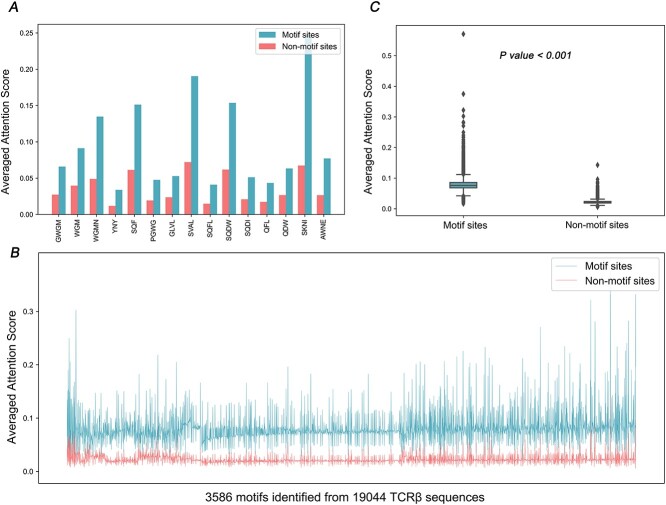
TCR-BERT highlights important residues for TCR-pMHC recognition through self-attention mechanisms. Panel **A** compares the averaged attention scores of residues within and outside motifs (non-motif) of the TCRs from the top 15 motif clusters with the highest GLIPH2 confidence scores. X-axis tick values are identified motif residues. Panel **B** displays the averaged attention scores of TCRs for all 3586 motif clusters. The x-axes in Panels A and B are sorted by the GLIPH2 confidence scores of the motif clusters. In Panel **C**, the box plot highlights the statistically significant difference in averaged attention scores between motif and non-motif residues. The *P*-value was determined by the Wilcoxon signed-rank test.

The success of our TABR-BERT model in predicting TCR-pMHC interactions was attributed to the capabilities of its underlying BERT-based embedding models (Figure 1A and B). The representation learning in BERT effectively captured the importance of amino acids in 1-dimensional sequences and key residue interactions critical for epitope and MHC binding. To gain deeper insights into the learning process of these embedding models, we conducted tests to demonstrate that (1). the residue attention scores from BERT's self-attention module pinpointed the crucial residues in TCR-pMHC recognition and that (2). BERT embeddings successfully captured epitope-MHC binding information.

#### TCR-BERT's embedding model, via its self-attention mechanism, adeptly emphasized pivotal residues within TCR sequences crucial for TCR-pMHC recognition

We demonstrated a significant association between residues' attention scores and their functional motifs. Our study utilized a dataset of 19 044 unique TCRβ sequences, with motifs identified using the GLIPH2 [39] algorithm. Particularly, residues within motifs exhibited substantially higher attention scores compared to those outside motifs of the TCRβ sequences from the top 15 motif clusters with the highest GLIPH2 confidence scores (Figure 4A). This trend persisted consistently across TCRβ sequences from all motif clusters (Figure 4B), with statistical significance (Figure 4C, *P*-value < 0.001). The results underscored the successful capture of potentially functional important residues through the TCR-BERT embedding model, thereby supporting the efficacy of the downstream transfer learning.

**Figure 5:**
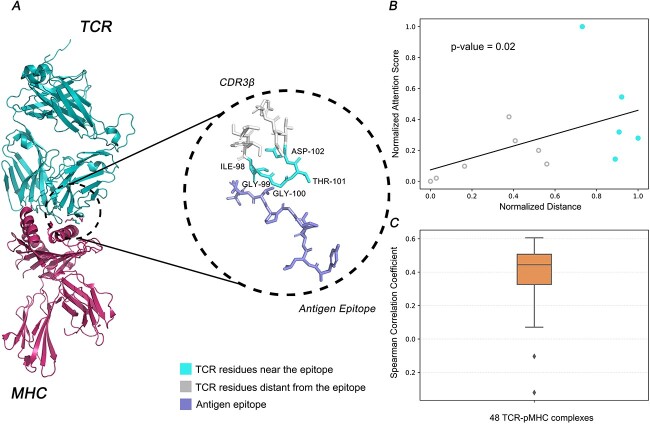
TCR-BERT identifies structurally important residues in TCR-pMHC complexes. Panel **A** illustrates the complex structure of a TCR binding to an HLA-A*0201 restricted epitope, glycoprotein 100 (gp100) ^280–288^ (PDB ID: 5EU6). The residues ILE-98, GLY-99, GLY-100, THR-101 and ASP-102, marked in cyan, demonstrate elevated attention scores and close proximity to the antigen epitope in the zoom-in plot. Other residues in TCR sequences are colored in gray. Panel **B** shows the scatter plot depicting the normalized averaged distance versus normalized averaged attention score for each amino acid within the TCR sequences, with a linear fitted line. The solid points indicate the five residues in red in Panel A. (See details of normalization methods in Supplementary Method 4.4 available online at http://bib.oxfordjournals.org/.) In Panel **C**, the box plot displays the distribution of the Spearman correlation coefficients between normalized distance and attention score for 48 TCR-pMHC complexes with available PDB structures. The mean and median of correlation coefficient is 0.383 and 0.433, respectively.

Moreover, the TCR-BERT embedding model underscored structurally important residues in TCR sequences. We showed that the residues proximal to binding epitopes in TCR-pMHC interactions exhibited high attention scores. Taking the structure of the complex structure of a TCR binding to an HLA-A*0201 restricted epitope, glycoprotein 100 (gp100)^280–288^ (PDB ID: 5EU6) as an example (Figure 5A), residues ILE-98, GLY-99, GLY-100, THR-101 and ASP-102, which exhibited large attention values, were all adjacent to the epitope. The Spearman correlation coefficient of 0.603 (*P*-value = 0.02, calculated by a permutation test implemented in SciPy [40]) showed a strong correlation between the residues’ attention scores with their averaged distances to the epitope (Figure 5B). This pattern was consistent across 48 TCR-pMHC complexes with available structural data with the mean and the median Spearman correlation coefficient of 0.383 and 0.433, respectively (Figure 5C). These findings validated that TCR-BERT embedding effectively prioritizes residues crucial for TCR-pMHC interactions from a structural perspective.

#### The pMHC-BERT embedding model proficiently acquired epitope-MHC-I binding patterns and highlighted key residues within epitope sequences essential for TCR interactions

**Figure 6:**
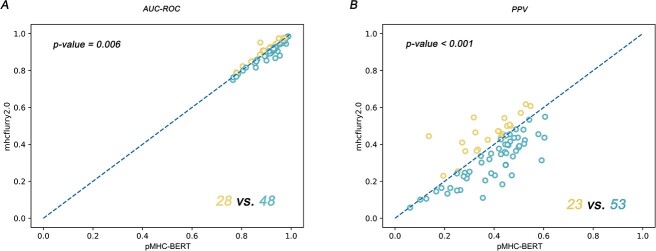
pMHC-BERT effectively captures the epitope and MHC binding information. In Panel **A**, pMHC-BERT with an MLP predictor outperforms MHCflurry2.0 in 48 out of 76 test samples within the Te-pMHC set, based on the AUC-ROC performance metric. In Panel **B**, our predictor demonstrates superior performance in 53 out of 76 test samples within the Te-pMHC set for the PPV metric. The blue points indicate cases where pMHC-BERT exhibits higher performance metrics than MHCflurry2.0. *P*-values were determined using the Wilcoxon signed-rank test.

The pMHC-BERT model was applied to predict epitope-MHC-I interactions using an independent test set, Te-pMHC. The results demonstrated competitive performance compared to established epitope-MHC-I predictors, such as MHCflurry2.0 [31], netMHCpan4.1 [28] and MixMHCpred2.2 [41]. Our method exhibited significantly higher AUC-ROC values and positive predictive values (PPV) compared to MHCflurry2.0, as depicted in Figure 6A and B, with both *P*-values <0.01. Moreover, it showed comparable results to netMHCpan4.1 and MixMHCpred2.2. (See detailed results comparing our method to netMHCpan4.1 and MixMHCpred2.2 in Supplementary Method 4.5 available online at http://bib.oxfordjournals.org/.)

Subsequently, we demonstrated that epitope residues in proximity to binding TCRs in TCR-pMHC complexes exhibited elevated attention scores akin to TCR residues. In the case of the LS01-TCR/M1-HLA-A*02 complex (PDB ID: 5ISZ), we calculated the Spearman correlation coefficient between epitope residue attention scores and their average distances to the TCR, yielding a strong positive correlation coefficient of 0.8. This pattern remained consistent across 48 TCR-pMHC complexes, with a mean and median Spearman correlation coefficient of 0.325 and 0.340, respectively.

Overall, the success of TABR-BERT stemmed from the powerful TCR-BERT and pMHC-BERT. Leveraging the extensive TCR sequence, epitope sequence and epitope-MHC binding data effectively mitigated data scarcity issues in TCR-pMHC interaction prediction and resulted in superior performance for our model. Finally, we employed our model to identify neoantigen-reactive TCRs, exemplifying its potential application in TCR-based cancer immunotherapy.

#### TABR-BERT identified TCRs responsive to neoantigens arising from hotspot TP53 mutations

**Figure 7:**
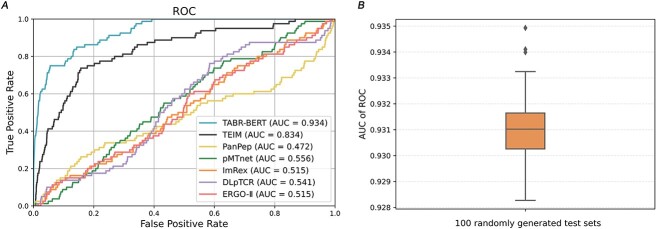
TABR-BERT identified neoantigen-reactive TCRs from hotspot TP53 mutations. Panel **A** illustrates the ROC curve depicting the prediction performance of all models for the neoantigen-reactive TCR dataset. Panel **B** displays the boxplot representing the distribution of AUC-ROCs calculated from 100 diverse test sets.

TP53 is a pivotal tumor suppressor gene frequently mutated in various cancers at rates from 38% to 50% in ovarian, esophageal, colorectal, head and neck and lung cancers [42]. We applied the TABR-BERT model to identify TCRs targeting MHC-I restricted neoantigens from eight hotspot TP53 mutations, encompassing 3.717% of TP53-mutated patients. Shown in Figure 7A, TABR-BERT demonstrated the highest performance with an AUC-ROC of 0.934, surpassing the second-best performer TEIM (AUC-ROC 0.834). For six out of eight hotspot mutations, TABR-BERT identified at least one neoantigen-reactive TCR among the top 10 hits. (See the positive prediction values (PPV) for top 10, 20 and 30 highest prediction scores for each hotspot mutations in Supplementary Table 4 available online at http://bib.oxfordjournals.org/.) We demonstrated the consistent performance of TABR-BERT across 100 diverse test sets, each with randomly selected negative cases, as indicated by the median value of 0.931 in Figure 7B. Notably, this application also showcased the model's ability to make predictions in unseen scenarios, as all neoantigens were distinct from the training set. (See details of test results in Supplementary Table 5 available online at http://bib.oxfordjournals.org/.)

## CONCLUSION AND DISCUSSION

Computational identification of TCR-pMHC interactions holds significant potential for advancing immunotherapy development. It is particularly important in the context of personalized treatments involving unseen antigen epitopes (‘zero-shot’ setting), such as neoantigens for cancer vaccines and TCR-engineered T cell (TCR-T) therapies [43]. The zero-shot prediction scenario for unseen epitopes remains challenging due to the cross-reactivity of TCR-pMHC recognition patterns [44]. While deep learning models like CNNs and long-short term memory (LSTM) networks have shown encouraging progress, most current approaches rely on scarce TCR-pMHC interaction data. Training datasets often have less than 1 million data points [30], obtained through expensive and time-consuming experimental methods. Such small training sets may lead to overfitting and limited prediction performance in complex deep learning architectures [45]. In contrast, vast amounts of unlabeled TCR sequences (over 100 million) and pMHC binding data (over 4 million) are available in databases such as TCRdb [20] and IEDB [30]. Therefore, the development of pre-trained models to capture functionally relevant information from these data, coupled with the application of transfer learning for TCR-pMHC interaction prediction, presents a promising opportunity to enhance prediction performance.

We used BERT architectures to extract valuable information from TCR sequences, epitope sequences, MHC subtypes and pMHC binding data. BERT has potent representation learning capabilities through self-attention mechanisms. It has been successfully applied in various biological research areas, including large-scale protein sequence representations [46].

In our model, TCR-BERT effectively identified crucial residues within unlabeled TCR sequences for TCR-pMHC interactions via self-attention mechanisms. Likewise, pMHC-BERT captured epitope and MHC binding information through the NSP task. We acknowledge that we have not fully elucidated the inner workings of the BERT model ('black box'). However, we confidently assert that the utilization of these pre-trained representations significantly contributes to our model's exceptional performance in predicting TCR-pMHC interactions. Additionally, our model demonstrated superior predictive capability compared to pMTnet, particularly when dealing with previously unseen epitopes. This observation underscores the efficiency of BERT-based embedding models in capturing critical information from TCR and pMHC data, as compared to autoencoder and LSTM methods employed in pMTnet.

In conclusion, TABR-BERT efficiently represents the extensive TCR sequence and pMHC complex data, resulting in superior TCR-pMHC interaction prediction, particularly for unseen epitopes. Our ongoing efforts focus on further enhancing its performance by incorporating meta-information through different learning tasks in pre-training and explicitly integrating TCR-epitope-MHC interaction details into the prediction model.

**Key Points**

- We developed a novel deep learning model for predicting TCR-pMHC interactions, TABR-BERT, which incorporates BERT-based transfer learning to effectively handle data scarcity for TCR-pMHC binding prediction.
- TABR-BERT consistently outperforms state-of-the-arts models across multiple benchmark sets, demonstrating its enhanced predictive accuracy and generalization capability, especially for unseen epitopes.
- Leveraging vast unlabeled TCR and pMHC data, TCR-BERT and pMHC-BERT, the underlying embedding models of TABR-BERT, successfully extract relevant information in TCR sequences and binding patterns of epitopes and MHC molecules, contributing to the model's predictive success.
- TABR-BERT serves as a valuable tool for investigating TCR-pMHC interactions and advancing immunotherapies.

## Supplementary Material

new-tabr-bert-supplementarydata-revision-bib_bbad436

new-tabr-bert-supplementarytables-revision-bib_bbad436

## Data Availability

TABR-BERT is implemented in Python. The software is freely accessible at the GitHub repository (https://github.com/Freshwind-Bioinformatics/TABR-BERT). The training and benchmark datasets used in the study are derived from public sources and are accessible from https://zenodo.org/record/8215354. Supplementary Table 5 available online at http://bib.oxfordjournals.org/ is accessible from https://zenodo.org/record/8412277.

## Author Biographies

**Jiawei Zhang** is an Assistant Bioinformatics R&D Engineer at Fresh Wind Biotechnologies Inc. (Tianjin). His research focuses on large language model and its applications in immunotherapy.

**Wang Ma** is a Bioinformatics R&D Engineer at Fresh Wind Biotechnologies Inc. (Tianjin). His research focuses on artificial intelligence and bioinformatics.

**Hui Yao**, Ph.D., is a Senior Vice President for Informatics at Fresh Wind Biotechnologies USA Inc. His research focuses on machine learning and deep learning methodology, computational biology, cancer biology and immunotherapy.
