# Supplementary material for: Accurate TCR-pMHC interaction prediction using a BERT-based transfer learning method
Source: Brief Bioinform. 2023 Dec 1;25(1):bbad436. doi: 10.1093/bib/bbad436 (PMC10783865; doi:10.1093/bib/bbad436)
Supplement: new-tabr-bert-supplementarydata-revision-bib_bbad436 [file new-tabr-bert-supplementarydata-revision-bib_bbad436.docx]

Content

[Supplementary Method 1 2](#_Toc30103)

[1.1 Preprocessing TCR sequences 2](#_Toc4680)

[1.2 Training TCR-BERT 2](#_Toc16742)

[Supplementary Method 2 3](#_Toc17707)

[2.1 Preprocessing pMHC sequences 3](#_Toc25712)

[2.2 Training pMHC-BERT 3](#_Toc11543)

[Supplementary Method 3 5](#_Toc11571)

[3.1 Constructing the master TCR-pMHC data set. 5](#_Toc30295)

[3.2 Producing negative cases for TCR-pMHC training and prediction 5](#_Toc30576)

[3.3 Training TCR-pMHC prediction model 6](#_Toc29641)

[3.4 Computing percentile rank for prediction 6](#_Toc63)

[Supplementary Method 4 7](#_Toc5389)

[4.1 Computing attention scores of TCR sequences and of epitope sequences 7](#_Toc11266)

[4.2 Collecting the motifs, identified by GLIPH2, of TCR sequences 7](#_Toc6967)

[4.3 Calculating the averaged distances between TCR residuals to their binding epitopes, as well as between epitope residues and their corresponding TCRs. 8](#_Toc21713)

[4.4 Normalization methods used for comparison of attention score and distance 9](#_Toc26630)

[4.5 Testing pMHC-BERT as an epitope-MHC-I binding predictor 9](#_Toc11596)

[Supplementary Method 5: Benchmark Software 11](#_Toc25588)

[Supplementary Analysis 12](#_Toc25340)

[6.1 The influence of the hyperparameters 12](#_Toc6551)

[6.2 Detailed Description of the TCR-pMHC prediction data sets and performance breakdown 13](#_Toc17568)

[6.3 The influence of the size of training data 15](#_Toc22822)

[6.4 The influence of high-frequency ("hot”) epitope in the training set 16](#_Toc5155)

[6.5 The influence of epitope sequence similarity between the training and test sets 16](#_Toc3369)

[6.6 Prediction performance of unseen TCRs 18](#_Toc10515)

[6.7 Positive correlation between epitope residues’ attention scores and their distances from TCRs 19](#_Toc29077)

[Reference 22](#_Toc28605)

# Supplementary Method 1

## 1.1 Preprocessing TCR sequences

The data set, Tr-TCR, consists of 113,529,384 unique TCR CDR3β sequences downloaded from TCRdb [1]. The lengths of these TCRs were all 10-30, which accounted for 99.9% of all TCRdb data. In Tr-TCR, 25% of the amino acids in each TCR sequence were randomly masked. Specifically, 80% of the masked amino acids were replaced with masked tokens, 10% with randomly selected alternative amino acids, and 10% remained unchanged. Subsequently, the masked sequences were padded to a fixed length of 30 using padding tokens as input data. The utilization of padding tokens was instrumental in maintaining consistent input dimensions, leading to improved computational efficiency and effective training on sequence inputs with variable lengths.

## 1.2 Training TCR-BERT

The validation set comprises 200,000 preprocessed inputs randomly chosen from Tr-TCR, and the remaining data serves as the training set. During each epoch, 1,000,000 training samples were randomly drawn with replacement. Training utilized masked language modeling (MLM) task [2] with a cross-entropy loss [3]. We conducted a grid search [4], exploring a predefined set of hyperparameters guided by validation loss.

# Supplementary Method 2

## 2.1 Preprocessing pMHC sequences

The data set, Tr-pMHC, consists of 4,128,332 pMHC pairs download from IEDB, including 179,801 binding affinity (BA) data and 3,948,531 mass spectrometry (MS) data. Epitope sequences and MHC pseudo-sequences [5] underwent the same mask preprocessing as TCR sequences. The resulting masked epitope sequence and MHC pseudo-sequence were concatenated with segment tokens in between, and then padded to a length of 55, considering the length of 37 for MHC pseudo-sequences and 16 for the longest epitope sequences. This yielded the pMHC sequence for input, with segment tokens facilitating segmentation between MHC pseudo-sequences, epitope sequences, and padding tokens.

## 2.2 Training pMHC-BERT

Tr-pMHC was divided into training and validation sets in the ratio of 8:2, where the validation set was preprocessed once at the beginning of training and was not changed in the subsequent training process. The training set was preprocessed once again at the beginning of each epoch to make the tokens that were masked in the pMHC sequence different in each epoch of training and increased the richness of the training data.

The model was trained using two tasks: Selective Mask Language Modeling (SMLM) and Next Sentence Prediction (NSP) [2]. In the SMLM task, the model predicted masked amino acids specifically when the input epitope and MHC could bind, employing cross-entropy loss. Meanwhile, the NSP task focused on predicting epitope-MHC binding using mean square error (MSE) loss [3]. Both losses were combined to compute the overall loss function. The incorporation of the SMLM task aimed to alleviate interference and prevent unwanted information learning during training. For the NSP task, the binding affinity data was transformed to a 0-1 scale using the formula [5],$1-{log}_{50000}(nM affinity)$, enabling training with MS data. Like TCR-BERT, we performed a grid search based on predefined hyperparameters guided by validation loss.

# Supplementary Method 3

## 3.1 Constructing the master TCR-pMHC data set.

We collected data from McPAS [6] including 39,985 TCR-pMHC pairs and PIRD [7], including 51,139 TCR-pMHC pairs. From the IEDB database [8], we obtained 197,229 TCR-pMHC pairs using specific filtering parameters, “Linear Epitope”, “Has Receptor Sequence”, “Receptor Type TCR αβ”, “Positive Assays Only”, “T Cell Assays”, “MHC Class I”, “Humans”, “Any Disease” and “Any Reference Type”. Additionally, 71,799 TCR-pMHC pairs were obtained from the VDJDB [9] database, using the filtering criteria, “Species Human”, “TRB” and “MHC1”. After merging four data sets and removing duplicates, we obtained 71,836 unique positive TCR-pMHC pairs as the master TCR-pMHC data set, comprising 64,967 unique CDR3β sequences, 624 unique epitopes, and 64 unique MHCs.

## 3.2 Producing negative cases for TCR-pMHC training and prediction

We employed a healthy TCR dataset comprising 60,333,379 TCRs [10,11], obtained from 587 healthy individuals' peripheral blood samples without prior exposure to known antigens. This dataset was divided into three subsets: Tr-healthy, Te-healthy, and Bg-healthy (background healthy set). These subsets were utilized for generating negative examples in the training set, benchmark testing sets (Te-S1, Te-S2, Te-S3, Te-S4 and TCRs targeting hotspot TP53 mutations data set), and as a background distribution for calculating percentile ranks, respectively. Negative cases in all sets were generated by pairing the pMHC in positive cases with randomly selected healthy TCRs from Tr-healthy, Te-healthy, and Bg-healthy, respectively.

## 3.3 Training TCR-pMHC prediction model

The Tr-TCRpMHC dataset was split into training and validation sets with a 9:1 ratio. Negative cases in the validation set were kept unchanged throughout the training process. On the other hand, in the training set, negative examples were regenerated every 5 epochs to augment the data and enhance the training process. We performed a grid search for hyperparameter selection guided by validation loss.

## 3.4 Computing percentile rank for prediction

To determine the percentile rank for a given TCR-pMHC pair during prediction, we created a background distribution comprising 1000 TCR-pMHC pairs with the original pMHC and 1000 randomly selected TCRs from the Bg-healthy set. The percentile rank of the given TCR-pMHC pair was then calculated as its MLP output's percentile within the background distribution. The final rank score was obtained by subtracting the percentile rank from 1, ensuring that a higher rank score indicated a higher likelihood of TCR-pMHC binding. To assess the impact of background ensemble size, we computed performance metrics on the benchmark test sets across various sizes: 1,000, 2,000, 5,000, and 10,000. Supplementary Table 6 presented the results, highlighting robust performance. For computational efficiency, we selected a size of 1,000.

# Supplementary Method 4

## 4.1 Computing attention scores of TCR sequences and of epitope sequences

The attention scores of TCR amino acids were calculated using attention matrices extracted from the four Transformer encoder blocks [12] in the TCR-BERT architecture. For each input TCR sequence, each encoder block generated a 3-D array with dimensions of 8 attention heads x 30 (length of TCR with padding) x 30 (length of TCR with padding). To obtain the attention scores, we first averaged the 3-D arrays across the 8 heads, resulting in 30x30 matrices. These matrices were then multiplied across the four blocks. Finally, we performed column-wise averaging and removed zero-padding values to obtain the attention scores for each amino acid.

Utilizing an identical computational approach for TCR sequences, we computed attention scores for epitope amino acids based on the attention matrices extracted from the four Transformer encoder blocks within the pMHC-BERT architecture.

## 4.2 Collecting the motifs, identified by GLIPH2, of TCR sequences

We obtained a dataset containing 19,044 unique TCRβ sequences from 58 individuals [13]. The GLIPH2 algorithm [13] was used to identify motifs, resulting in the clustering of TCRβ sequences into 3,586 motif groups representing TCRs with shared recognition of the same epitope and HLA restriction. GLIPH2 employed the Fisher-exact test to calculate confidence scores for each identified motif cluster.

## 4.3 Calculating the averaged distances between TCR residuals to their binding epitopes, as well as between epitope residues and their corresponding TCRs.

The minimal distance between heavy atoms was employed to measure the proximity between two amino acids. Supplementary Figure 1 illustrates the distance map of TCR residues and epitope residues in the complex structure of a TCR binding to a HLA-A*0201 restricted epitope, glycoprotein 100 (gp100) ^280–288^ (PDB ID: 5EU6). The averaged CDR3β amino acid distance to all residues in the epitope was calculated to represent the CDR3β's amino acid distance from the epitope. Similarly, we computed the average amino acid distance of an epitope residue to all CDR3β residues to assess the epitope's proximity to CDR3β. This computation utilized Biopython [14], a Python package for computational biology and bioinformatics.


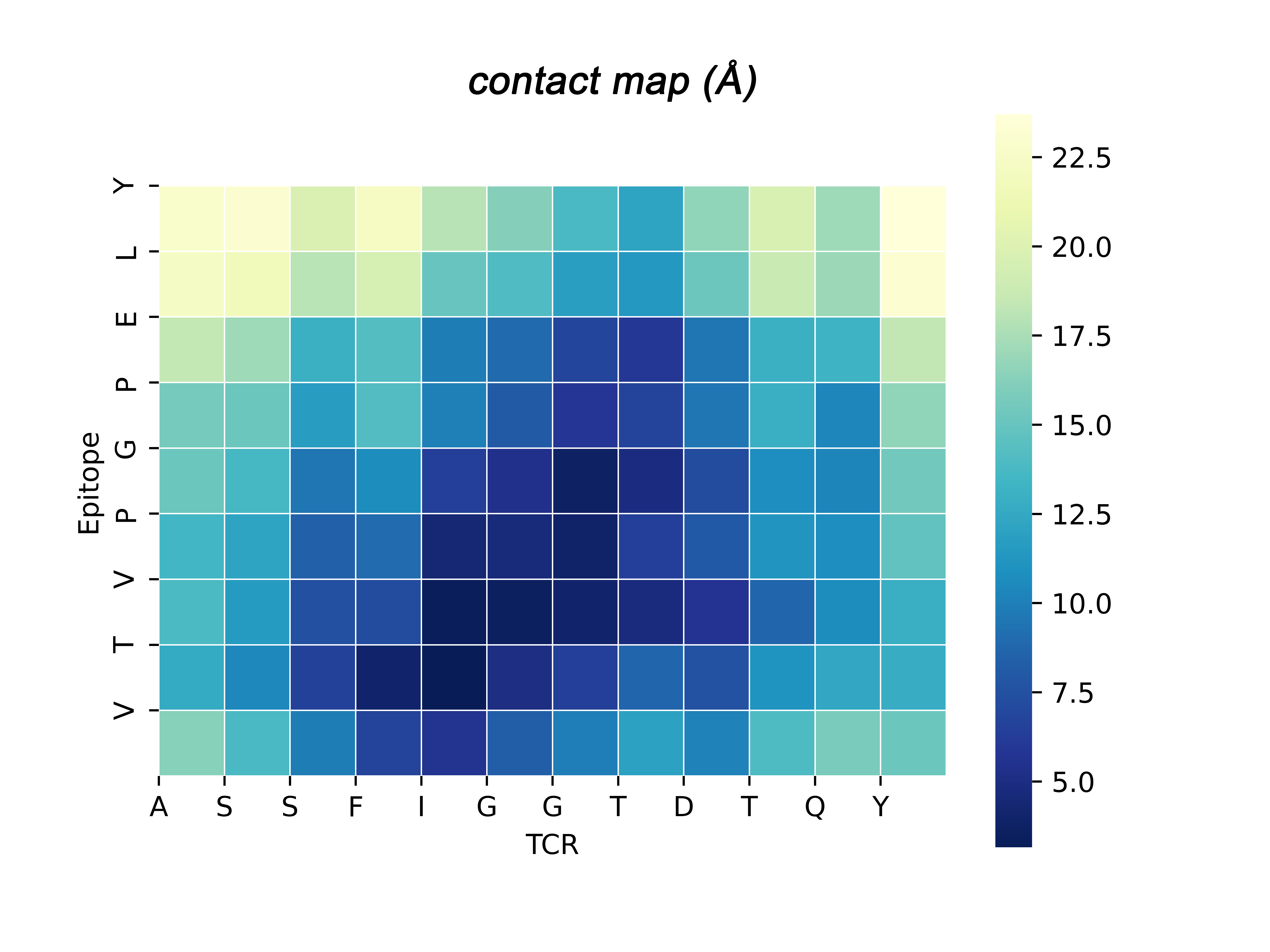


**Supplementary Figure 1.** **The distance map between residues in TCR and those in the epitope in the complex structure of a TCR binding to the gp100^280–288^ (PDB ID: 5EU6).**

## 4.4 Normalization methods used for comparison of attention score and distance

The attention score was normalized by the formula:

$$A_{i}^{N}= \frac{A_{i}-A^{\mathrm{MIN}}}{A^{\mathrm{MAX}}-A^{\mathrm{MIN}}}$$

and the averaged distance is normalized by formula:

$$D_{i}^{N}= \frac{D^{\mathrm{MAX}}-D_{i}}{D^{\mathrm{MAX}}-D^{\mathrm{MIN}}}$$

where $\boldsymbol{A}$ and $\boldsymbol{D}$ represent the attention score and the averaged distance to its binding epitope for each amino acid in TCR sequences, and $\boldsymbol{A}_{\boldsymbol{i}}$ and $\boldsymbol{D}_{\boldsymbol{i}}$ represent the amino acids currently considered. $MAX$ and $MIN$ represent the maximum and minimum values for the TCR sequence, respectively.

## 4.5 Testing pMHC-BERT as an epitope-MHC-I binding predictor

pMHC-BERT was utilized as an epitope-MHC-I binding predictor during the NSP task training. Specifically, the training process involved converting the 55x256 embedding matrix from pMHC-BERT into a one-dimensional vector of length 256 through column-wise summation. This vector served as the input for a multi-layer perceptron (MLP) classifier, consisting of a dense layer of 64 neurons activated by SELU function [15], followed by dropout layers with a rate of 0.2. The final layer of the MLP classifier comprised a single neuron with sigmoid activation [3]. We assessed the prediction accuracy of pMHC-BERT for epitope-MHC-I binding without any additional training. The evaluation was conducted in comparison to MHCflurry2.0 [5] netMHCpan4.1 [16] and MixMHCpred2.2 [17], using an independent multi-allele test set of 9,158,100 peptide-MHC-I pairs. For performance assessment, we used the positive predictive value (PPV) and the area under the Receiver Operating Characteristic curve (AUC-ROC). PPV was determined as the ratio of true positives among the top 1% predicted scores per sample. The choice of 1% was made to balance the considerable variance in the number of true positives across the samples.

The Supplementary Table 7 presented a summary of the testing outcomes, encompassing both operational models of netMHCpan4.1, namely Binding Affinity (BA) and Mass-Spectrometry Eluted Ligands (EL). In terms of the AUC-ROC metric, pMHC-BERT exhibited superior performance compared to all other models. Concerning PPV at 1%, it outperformed MHCflurry 2.0 and NetMHCpan4.1.BA but did not surpass the performance of NetMHCpan4.1.EL and MixMHCpred2.2.

# Supplementary Method 5: Benchmark Software

During benchmark testing, we employed various software with specific settings. For TEIM [18], we used the "seq inference" model with default parameters for prediction. It is worthy to note that TEIM can only input epitopes of lengths 8-12 and TCR CDR3 sequences of lengths up to 20. pMTnet [19] was run with its default parameters for prediction. ImRex [20] was run with its default parameters for prediction. ERGO-II [21] utilized a model trained by the VDJdb dataset, and feature configurations were set to MHC-I for prediction. PanPep [22] was used in "zero-shot" mode (not fine-tuned during prediction) with default parameters for prediction. DLpTCR [23] was set to "β chain" mode with default parameters for prediction.

# Supplementary Analysis

## 6.1 The influence of the hyperparameters

**6.1.1 TCR-BERT and pMHC-BERT**

We conducted an extensive evaluation of alternative hyperparameters in two pre-trained model, including learning rates (0.00001, 0.0001, 0.0005), batch sizes (256, 1024), attention head numbers (4, 6, 10, 12), transformer encoder block counts (2, 6), and embedding layer sizes (128, 512). Supplementary Table 8A presented our findings, demonstrating that the TABR-BERT model exhibits robustness to hyperparameter choices, evidenced by AUC-ROC values spanning from 0.821 to 0.842 and AUC-PR values ranging from 0.828 to 0.879 for the largest test set (Te-S1). Notably, a tenfold escalation in the learning rate to 0.0005 represented the sole exception, leading to a substantial reduction in both AUC-ROC and AUC-PR to 0.490 and 0.493, respectively.

**6.1.2 TCR-pMHC prediction model**

We conducted a comprehensive assessment of the influence of hyperparameters of model training and architecture in the TCR-pMHC prediction model. Our investigation revealed that variations in model training, including learning rates (0.0001, 0.00001) and batch sizes (128, 512), had negligible effects on model performance.

The TCR-pMHC prediction model comprises two key components: a low-rank adaptation (LoRA) [24] segment, responsible for rank reduction of TCR and pMHC embeddings, and a multilayer perceptron (MLP) predictor. In the LoRA segment, the original model transformed 54x256 (pMHC embedding) and 30x256 (TCR embedding) vectors into four vectors: 54x1, 256x1, 30x1, and 256x1. We explored three mapping configurations: 'larger mapping,' doubling vector lengths to 108x1, 512x1, 60x1, and 512x1; 'length mapping,' only maintaining lengths at 54x1 and 30x1; and 'embedding dimensional mapping,' yielding 256x1 and 256x1 vectors. As a result, the model's predictive performance exhibited robustness to LoRA configurations, as evidenced by AUC-ROC values ranging from 0.826 to 0.841 and AUC-PR values spanning 0.843 to 0.872 for Te-S1.

Regarding the MLP layer, we investigated 'larger MLP,' comprising three dense layers with 300, 200, and 100 neurons, respectively. Conversely, 'smaller MLP' consisted of a dense layer with 100 neurons. Test results indicated that 'larger MLP' yielded comparatively inferior outcomes, with an AUC-ROC of 0.810 and AUC-PR of 0.784 for Te-S1, possibly attributable to overfitting stemming from an excessive number of model parameters. In sum, our findings underscored the robustness of the model's performance to hyperparameters and architectural choices (See detailed results for each test set provided in Supplementary Table 8B).

## 6.2 Detailed Description of the TCR-pMHC prediction data sets and performance breakdown

We illustrated the length distributions of TCR CDR3β sequences in both the training set (Tr-TCR-pMHC) and the test sets (Te-S1, Te-S2, Te-S3, Te-S4) and the distributions of clonotype abundance in Supplementary Figures 2 and 3, respectively. Performance analysis categorized sequences into three groups: short (length < 13), medium (length 13-16), and long (length >16), with detailed results provided in Supplementary Table 9A. Notably, long sequences exhibited suboptimal performance, exemplified by Te-S1 with AUC-ROC and AUC-PR values of 0.694 and 0.614. This might be primarily due to the limited representation of long CDR3β sequences, comprising only 8.02% of the Tr-TCR-pMHC dataset, resulting in insufficient training data for this category. Furthermore, the impact of outliers in small test sets can significantly influence performance metrics.

The prediction performance of epitope sequences was assessed based on epitope length. Supplementary Table 9B presented the results, demonstrating consistent prediction performance across various epitope lengths, except for Te-S3, where deviations were observed for epitope lengths of 8 and 13. Specifically, the AUC-ROC values were 0.662 and 0.726, while the AUC-PR values were 0.746 and 0.692, respectively. These deviations might be primarily attributed to the constrained size of the test sample. Moreover, an analysis of prediction performance concerning major MHC-I subtypes demonstrated similar consistency, as evidenced in Supplementary Table 9C.





**Supplementary Figure 2.** TCR CDR3β length distribution for different datasets.





**Supplementary Figure 3.** TCR clonotype abundance for different datasets.

## 6.3 The influence of the size of training data

To evaluate the impact of training data size, we conducted experiments where we progressively reduced the training set by randomly selecting 45,000, 20,000 and 10,000 out of 70,423 training data points. Supplementary Table 10 presented the results, revealing that the prediction performance remained robust across all four test sets, albeit with gradual decreases. For instance, in Te-S1, the largest zero-shot test set for TABR-BERT, the AUC-ROC decreased from 0.842 to 0.829, while the AUC-PR decreased from 0.879 to 0.870.

## 6.4 The influence of high-frequency ("hot”) epitope in the training set

To investigate the influence of high-frequency epitopes within our training dataset, we retrained our TCR-pMHC prediction model after exclusion of 1) the most frequently occurring epitope, "KLGGALQAK," which constituted 37.7% of the training data, or 2) the top five "hot" epitopes, collectively, representing 71.4% of the training dataset (as detailed in Supplementary Table 11). Our findings, as presented in Supplementary Table 12, consistently indicated the robustness of our model’s prediction performance across all test sets. Interestingly, we observed an improvement in performance in test sets Te-S2 and Te-S3 when excluding the "KLGGALQAK" epitope. This observation suggested that the overrepresentation of a specific epitope within the training data may introduce bias into the model.

## 6.5 The influence of epitope sequence similarity between the training and test sets

To assess the impact of sequence similarity between training and test sets, we conducted a comparison of epitope sequences in correctly and incorrectly predicted cases. First, we employed the Youden Index [25] to determine optimal thresholds, as shown in Figure 3A, for distinguishing correct (true positive and negative cases) from incorrect (false positive and negative cases) predictions.

Subsequently, we performed pairwise epitope alignments using the Needleman-Wunsch algorithm [26] and calculated the alignment-length-normalized sequence similarity after removing duplicated sequences. The computation was carried out using the BLOSUM62 matrix [27] and the function pairwise2.align.globalds from the Biopython module. For each epitope in the test sets, we computed its average sequence similarity to all epitopes in the training set, considering this as its similarity to the training set.

Finally, we compared the sequence similarity to the training set between epitopes in correct and incorrect predictions, as illustrated in Supplementary Figure 4. Notably, the sequence similarity in incorrectly predicted cases was found to be statistically significantly higher than that of correctly predicted cases, with a p-value of 0.026 in Te-S1. No other statistically significant results were observed in other test sets. The p-value calculation was performed using the Wilcoxon rank-sum test, and detailed statistical information is available in Supplementary Table 3.





**Supplementary Figure 4.** Comparison of epitope sequence similarity between test and training data sets for correctly predicted vs. incorrectly predicted cases (P-values are calculated using the Wilcoxon rank-sum test).

## 6.6 Prediction performance of unseen TCRs

To evaluate the predictive capacity of our model on unseen TCRs, we conducted an experiment where we excluded 250 sequences overlapped with any of the test sets from the training data. Subsequently, we retrained the TCR-pMHC model using the remaining 70,173 TCR sequences.

The results, as depicted in Supplementary Table 8, indicated robust performance, albeit with a minor decrease. Specifically, the AUC-ROC and AUC-PR values changed from 0.842 and 0.879 to 0.829 and 0.871, respectively, for Te-S1. This demonstrates the model's ability to generalize to novel TCR sequences with only a marginal reduction in predictive accuracy. (See the details of the all test sets in Supplementary Table 13).

## 6.7 Positive correlation between epitope residues’ attention scores and their distances from TCRs

We investigated the relationship between epitope attention scores and their distances from TCRs in cases where complex structures were accessible. Using the structure of LS01-TCR/M1-HLA-A*02 complex (PDB ID: 5ISZ) as an example (Supplementary Figure 5A), we noted that residues LEU-3, GLY-4, PHE-5, VAL-6, PHE-7, and THR-8, which exhibited substantial attention values, were in close proximity to the TCR. This correlation was quantified using the Spearman correlation coefficient, yielding a robust value of 0.8 (p-value < 0.001), indicating a strong positive association between residue attention scores and their average distances from the TCR (Supplementary Figure 5B). This consistent pattern extended to 48 TCR-pMHC complexes, with a mean and median of Spearman correlation coefficient of 0.325 and 0.340, respectively (Supplementary Figure 5C).

We also investigated the relationship between epitope attention scores and their distance from MHC alpha chain in cases with accessible complex structures. As showed in Supplementary Figure 6, no significant correlation was observed between these two factors.


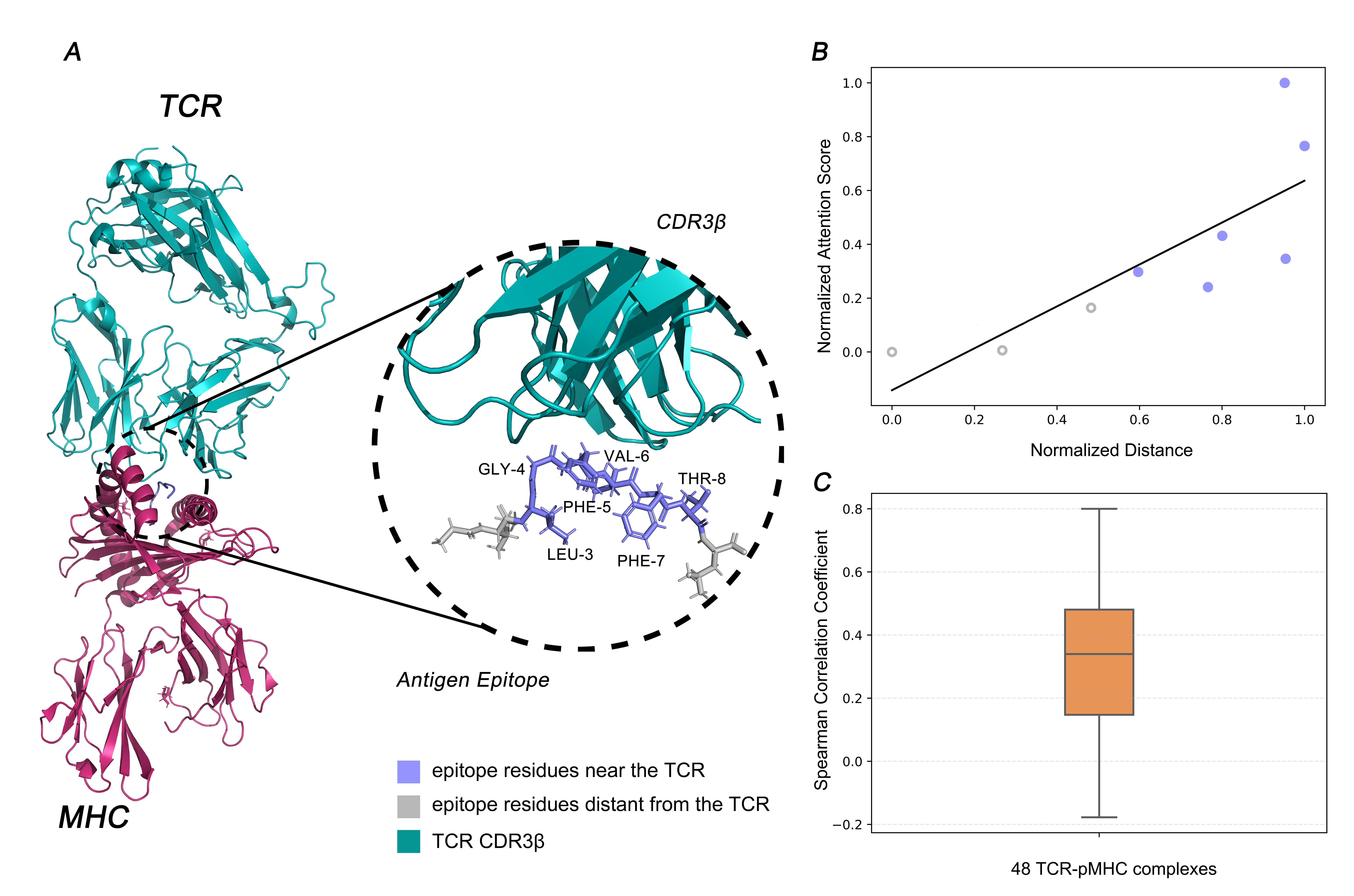


**Supplementary Figure 5.** **pMHC-BERT highlights the residues in epitopes important for TCR interaction.** Panel A: 3D structure of 5ISZ (PDB ID) with residues LEU-3, GLY-4, PHE-5, VAL-6, PHE-7, and THR-8 highlighted in violet due to elevated attention scores and proximity to the TCR. Panel **B** shows the scatter plot depicting the normalized averaged distance versus normalized averaged attention score for each amino acid within the epitope sequences, with a linear fitted line. The solid points indicate the six residues in violet in Panel A. In Panel **C**, the box plot displays the distribution of Spearman's correlation coefficients between normalized distances and attention scores for 48 TCR-pMHC complexes with available PDB structures. The mean and median of correlation coefficient is 0.325 and 0.340, respectively.


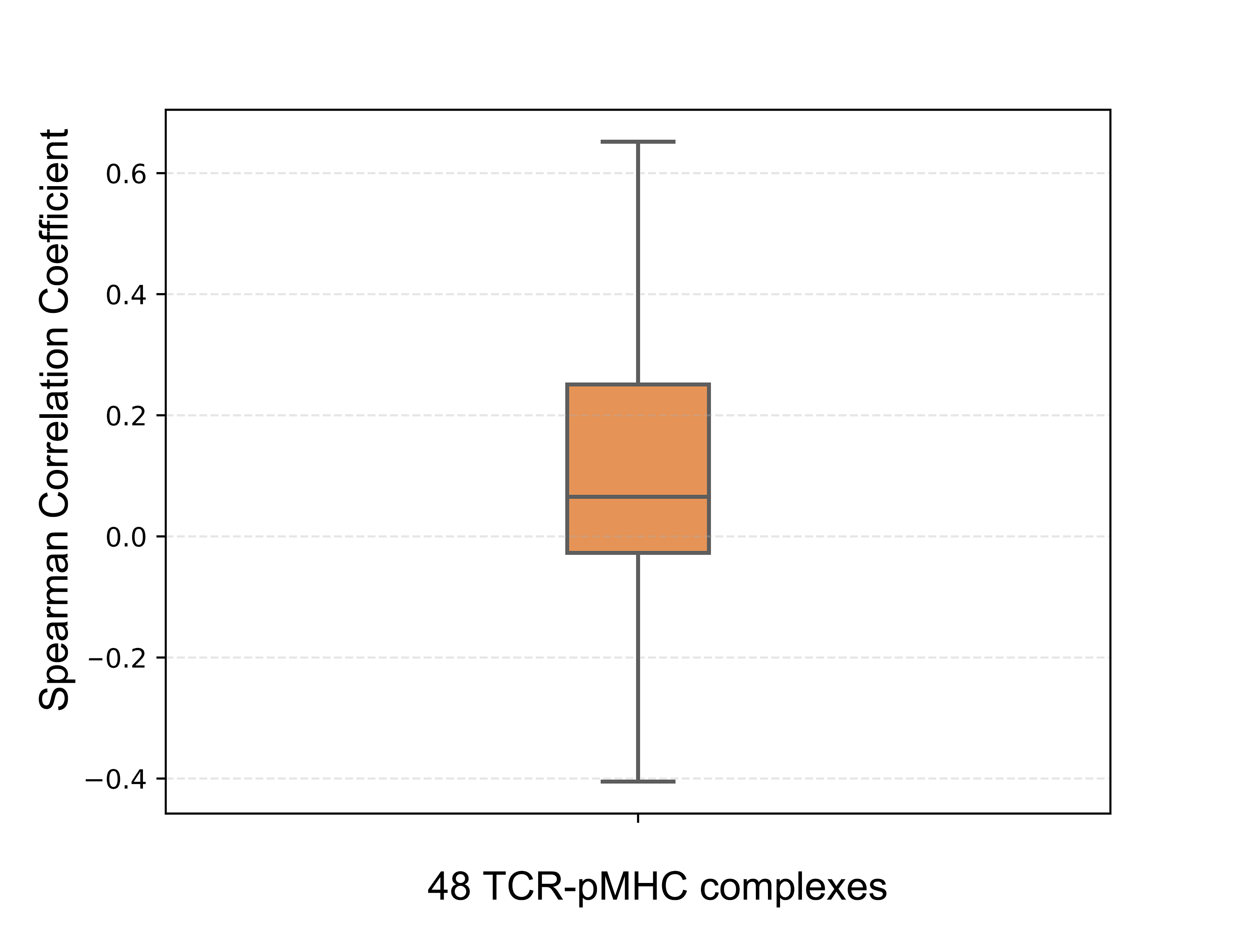


**Supplementary Figure 6** The box plot displays the distribution of Spearman's correlation coefficients between normalized distances between epitopes and MHC and epitope attention scores for 48 TCR-pMHC complexes with available PDB structures. The mean and median of correlation coefficient is 0.126 and 0.066, respectively.

# Reference

1. Chen S-Y, Yue T, Lei Q, et al. TCRdb: a comprehensive database for T-cell receptor sequences with powerful search function. Nucleic Acids Research 2021; 49:D468–D474

2. Devlin J, Chang M-W, Lee K, et al. BERT: Pre-training of Deep Bidirectional Transformers for Language Understanding. 2019;

3. Zhang A, Lipton ZC, Li M, et al. Dive into Deep Learning. 2023;

4. He X, Zhao K, Chu X. AutoML: A Survey of the State-of-the-Art. Knowledge-Based Systems 2021; 212:106622

5. O’Donnell TJ, Rubinsteyn A, Laserson U. MHCflurry 2.0: Improved Pan-Allele Prediction of MHC Class I-Presented Peptides by Incorporating Antigen Processing. cels 2020; 11:42-48.e7

6. Tickotsky N, Sagiv T, Prilusky J, et al. McPAS-TCR: a manually curated catalogue of pathology-associated T cell receptor sequences. Bioinformatics 2017; 33:2924–2929

7. Zhang W, Wang L, Liu K, et al. PIRD: Pan Immune Repertoire Database. Bioinformatics 2020; 36:897–903

8. Vita R, Mahajan S, Overton JA, et al. The Immune Epitope Database (IEDB): 2018 update. Nucleic Acids Res 2019; 47:D339–D343

9. Bagaev DV, Vroomans RMA, Samir J, et al. VDJdb in 2019: database extension, new analysis infrastructure and a T-cell receptor motif compendium. Nucleic Acids Research 2020; 48:D1057–D1062

10. Dean J, Emerson RO, Vignali M, et al. Annotation of pseudogenic gene segments by massively parallel sequencing of rearranged lymphocyte receptor loci. Genome Med 2015; 7:123

11. Luu AM, Leistico JR, Miller T, et al. Predicting TCR-Epitope Binding Specificity Using Deep Metric Learning and Multimodal Learning. Genes (Basel) 2021; 12:572

12. Vaswani A, Shazeer N, Parmar N, et al. Attention Is All You Need. 2023;

13. Huang H, Wang C, Rubelt F, et al. Analyzing the Mycobacterium tuberculosis immune response by T-cell receptor clustering with GLIPH2 and genome-wide antigen screening. Nat Biotechnol 2020; 38:1194–1202

14. Cock PJA, Antao T, Chang JT, et al. Biopython: freely available Python tools for computational molecular biology and bioinformatics. Bioinformatics 2009; 25:1422–1423

15. Klambauer G, Unterthiner T, Mayr A, et al. Self-Normalizing Neural Networks. 2017;

16. Jurtz V, Paul S, Andreatta M, et al. NetMHCpan-4.0: Improved Peptide-MHC Class I Interaction Predictions Integrating Eluted Ligand and Peptide Binding Affinity Data. J Immunol 2017; 199:3360–3368

17. Gfeller D, Schmidt J, Croce G, et al. Improved predictions of antigen presentation and TCR recognition with MixMHCpred2.2 and PRIME2.0 reveal potent SARS-CoV-2 CD8+ T-cell epitopes. cels 2023; 14:72-83.e5

18. Peng X, Lei Y, Feng P, et al. Characterizing the interaction conformation between T-cell receptors and epitopes with deep learning. Nat Mach Intell 2023; 5:395–407

19. Lu T, Zhang Z, Zhu J, et al. Deep learning-based prediction of the T cell receptor–antigen binding specificity. Nat Mach Intell 2021; 3:864–875

20. Moris P, De Pauw J, Postovskaya A, et al. Current challenges for unseen-epitope TCR interaction prediction and a new perspective derived from image classification. Briefings in Bioinformatics 2021; 22:bbaa318

21. Springer I, Tickotsky N, Louzoun Y. Contribution of T Cell Receptor Alpha and Beta CDR3, MHC Typing, V and J Genes to Peptide Binding Prediction. Frontiers in Immunology 2021; 12:

22. Gao Y, Gao Y, Fan Y, et al. Pan-Peptide Meta Learning for T-cell receptor–antigen binding recognition. Nat Mach Intell 2023; 5:236–249

23. Xu Z, Luo M, Lin W, et al. DLpTCR: an ensemble deep learning framework for predicting immunogenic peptide recognized by T cell receptor. Brief Bioinform 2021; 22:bbab335

24. Hu EJ, Shen Y, Wallis P, et al. LoRA: Low-Rank Adaptation of Large Language Models. 2021;

25. Youden WJ. Index for rating diagnostic tests. Cancer 1950; 3:32–35

26. Needleman SB, Wunsch CD. A general method applicable to the search for similarities in the amino acid sequence of two proteins. Journal of Molecular Biology 1970; 48:443–453

27. Henikoff S, Henikoff JG. Amino acid substitution matrices from protein blocks. Proceedings of the National Academy of Sciences 1992; 89:10915–10919

28. Haynes W. Wilcoxon Rank Sum Test. Encyclopedia of Systems Biology 2013; 2354–2355
