# Supplementary material for: Accurate TCR-pMHC interaction prediction using a BERT-based transfer learning method
Source: Brief Bioinform. 2023 Dec 1;25(1):bbad436. doi: 10.1093/bib/bbad436 (PMC10783865; doi:10.1093/bib/bbad436)
Supplement: new-tabr-bert-supplementarytables-revision-bib_bbad436 [file new-tabr-bert-supplementarytables-revision-bib_bbad436.docx]

**content**

[Supplementary Table 1 2](#_Toc13819)

[Supplementary Table 2 4](#_Toc10582)

[Supplementary Table 3 5](#_Toc8705)

[Supplementary Table 4 6](#_Toc26296)

[Supplementary Table 5 7](#_Toc22425)

[Supplementary Table 6 8](#_Toc9817)

[Supplementary Table 7 9](#_Toc31395)

[Supplementary Table 8A 10](#_Toc3604)

[Supplementary Table 8B 12](#_Toc27914)

[Supplementary Table 9A 14](#_Toc26043)

[Supplementary Table 9B 15](#_Toc22174)

[Supplementary Table 9C 16](#_Toc24576)

[Supplementary Table 10 17](#_Toc10469)

[Supplementary Table 11 18](#_Toc29574)

[Supplementary Table 12 19](#_Toc26209)

[Supplementary Table 13 20](#_Toc3464)

[References： 21](#_Toc28954)

# Supplementary Table 1

| Details of Te-pMHC | | | | |
| --- | --- | --- | --- | --- |
|  |  |  |  |  |
| sample_id | data_points | hla_subtypes | unique_epitopes | unique_epitopes_postive |
| 10-002-S1-TISSUE | 43100 | 6 | 21564 | 112 |
| 11-002-S1-TISSUE | 94600 | 6 | 54392 | 244 |
| 24616531 | 16700 | 6 | 10771 | 71 |
| 29/14-TISSUE | 404900 | 6 | 248572 | 1753 |
| 637/13-TISSUE | 238600 | 6 | 135169 | 786 |
| APHER1 | 149800 | 6 | 91457 | 751 |
| APHER6 | 32900 | 4 | 19983 | 112 |
| BCN-018-TISSUE | 93500 | 6 | 52663 | 157 |
| CD165 | 149800 | 6 | 89157 | 642 |
| CM467 | 345400 | 6 | 215120 | 1680 |
| CPH-07-TISSUE | 181600 | 6 | 104822 | 261 |
| CPH-08-TISSUE | 300800 | 6 | 180523 | 782 |
| CPH-09-TISSUE | 152700 | 6 | 85912 | 214 |
| FIB | 165600 | 6 | 99595 | 766 |
| FM-82 | 65500 | 6 | 38169 | 259 |
| FM-93/2 | 89900 | 6 | 51994 | 283 |
| GD149 | 343700 | 6 | 217542 | 1514 |
| HCC1143 | 64500 | 6 | 40849 | 372 |
| HCC1937 | 90100 | 6 | 58134 | 507 |
| HCT116 | 44900 | 6 | 29741 | 239 |
| HEK293 | 138900 | 6 | 88466 | 765 |
| HHC.2015 | 69800 | 5 | 44042 | 434 |
| HL-60 | 222100 | 6 | 147404 | 1529 |
| JY | 15600 | 6 | 10358 | 52 |
| JY.2015 | 42100 | 3 | 28324 | 249 |
| KESKIN_13240-002 | 12600 | 6 | 7426 | 33 |
| KESKIN_13240-005 | 101900 | 6 | 60275 | 205 |
| KESKIN_13240-006 | 55200 | 6 | 32378 | 232 |
| KESKIN_13240-015 | 124800 | 6 | 70736 | 475 |
| KESKIN_CP-594_V1 | 47600 | 6 | 31748 | 221 |
| KESKIN_DFCI-5283 | 99000 | 6 | 58711 | 487 |
| KESKIN_DFCI-5328 | 3800 | 6 | 2422 | 12 |
| KESKIN_DFCI-5341 | 12000 | 6 | 6562 | 58 |
| KESKIN_H4198_BT187 | 205500 | 6 | 125000 | 440 |
| KESKIN_H4512_BT145 | 191100 | 6 | 110489 | 408 |
| LEIDEN-004-TISSUE | 130800 | 6 | 73739 | 226 |
| LEIDEN-005-TISSUE | 206600 | 6 | 113594 | 427 |
| LNT-229 | 131900 | 3 | 81147 | 102 |
| LNT-229+DAC | 147700 | 3 | 89938 | 174 |
| MAVER-1 | 226500 | 6 | 139770 | 770 |
| MD155 | 74300 | 6 | 43185 | 241 |
| MEL-12 | 95900 | 3 | 55618 | 249 |
| MEL-15 | 584100 | 6 | 394275 | 3048 |
| MEL-16 | 311100 | 6 | 199069 | 1321 |
| MEL-624 | 37300 | 6 | 21431 | 99 |
| MEL-8 | 81900 | 6 | 52723 | 200 |
| MEWO | 49000 | 6 | 28000 | 114 |
| PAT_AC2 | 12400 | 4 | 7870 | 47 |
| PAT_C | 28700 | 4 | 17893 | 58 |
| PAT_CELG | 94200 | 6 | 57855 | 454 |
| PAT_CP2 | 17600 | 3 | 10650 | 61 |
| PAT_FL | 52700 | 4 | 33109 | 161 |
| PAT_J | 25600 | 4 | 14431 | 57 |
| PAT_JPB3 | 12900 | 4 | 7622 | 61 |
| PAT_JT2 | 17700 | 3 | 11120 | 73 |
| PAT_M | 13200 | 6 | 8263 | 26 |
| PAT_MA | 47300 | 6 | 26803 | 126 |
| PAT_ML | 21800 | 4 | 13652 | 57 |
| PAT_NS2 | 8500 | 3 | 5048 | 35 |
| PAT_NT | 34900 | 3 | 21367 | 102 |
| PAT_PF1 | 40600 | 6 | 23561 | 89 |
| PAT_R | 12300 | 6 | 7815 | 24 |
| PAT_RT | 69800 | 6 | 39050 | 257 |
| PAT_SR | 26000 | 4 | 14382 | 55 |
| PAT_ST | 4000 | 4 | 2943 | 19 |
| PD42 | 45700 | 6 | 29087 | 228 |
| RA957 | 599800 | 6 | 386034 | 2779 |
| RPMI8226 | 165700 | 6 | 105269 | 996 |
| SK-MEL-5 | 59800 | 4 | 37965 | 312 |
| T98G | 185800 | 3 | 111935 | 134 |
| T98G+DAC | 187600 | 3 | 112711 | 138 |
| THP-1 | 162100 | 6 | 102716 | 823 |
| TIL1 | 204200 | 5 | 123006 | 677 |
| TIL3 | 237100 | 6 | 150526 | 1134 |
| U-87 | 111200 | 3 | 66316 | 80 |
| U-87+DAC | 171200 | 3 | 106103 | 268 |

# Supplementary Table 2

| Anti-mutant p53 TCR library | | | | | |
| --- | --- | --- | --- | --- | --- |
|  |  |  |  |  |  |
| TCR source  (previous  reporting) | TP53 mutation | TP53 mutation  frequency (%) | HLA restriction | HLA  frequency (%) | Potentially  treatable  patient (%) |
| 4316 | C135Y | 0.426 | HLA-A:29:02 | 7.060 | 0.030 |
| 4350 | L111R | 0.011 | HLA-A:11:01 | 14.000 | 0.001 |
| 4356 | Q331H | 0.011 | HLA-B:40:01 | 11.000 | 0.001 |
| 4141 (10,15); 4196 (12) | R175H | 5.530 | HLA-A:02:01 | 47.400 | 2.621 |
| 4266 (10,15) | R248W | 3.218 | HLA-A:68:01 | 6.380 | 0.205 |
| 4324 | T211I | 0.032 | HLA-C:06:02 | 18.640 | 0.006 |
| 4259 | Y220C | 1.790 | HLA-A:02:01 | 47.400 | 0.848 |
| 4414 | Y220D | 0.011 | HLA-A:02:01 | 47.400 | 0.005 |
|  |  |  |  |  |  |
| TP53 mutation frequency: International Agency for Research on Cancer (IARC) TP53 Database [1] | | | | | |
| HLA frequency: Phenotype frequency of Caucasian populations in the United States; when the phenotype frequency is not available, twice the allele frequency is reported (http://www.allelefrequencies.net/) [2]. | | | | | |

# Supplementary Table 3

| statistical information about similarity of correctly predicted and incorrectly predicted epitopes to epitopes in the training set | | | | | |
| --- | --- | --- | --- | --- | --- |
|  |  |  |  |  |  |
|  |  | Te-S1 | Te-S2 | Te-S3 | Te-S4 |
| median | correct | 1.59556 | 1.59930 | 1.57568 | 1.61855 |
|  | incorrect | 1.62292 | 1.58536 | 1.56629 | 1.63167 |
|  |  |  |  |  |  |
| upper quartile | correct | 1.68723 | 1.68887 | 1.66667 | 1.69510 |
|  | incorrect | 1.71741 | 1.69685 | 1.66601 | 1.71829 |
|  |  |  |  |  |  |
| lower quartile | correct | 1.50131 | 1.46457 | 1.48712 | 1.50525 |
|  | incorrect | 1.52056 | 1.45445 | 1.45940 | 1.52318 |
|  |  |  |  |  |  |
| number of case | correct | 491 | 222 | 266 | 180 |
|  | incorrect | 289 | 96 | 136 | 52 |
|  |  |  |  |  |  |
|  | p-value | 0.02633 | 0.88853 | 0.50807 | 0.45712 |
|  |  |  |  |  |  |
| P-values were determined using the Wilcoxon rank-sum test. | | | | | |

# Supplementary Table 4

| PPV for each hotspot mutations | | | |
| --- | --- | --- | --- |
|  |  |  |  |
|  | PPV10 | PPV20 | PPV30 |
| C135Y | 0.200 | 0.250 | 0.167 |
| L111R | 0.000 | 0.000 | 0.067 |
| Q331H | 0.500 | 0.250 | 0.167 |
| R175H | 0.000 | 0.100 | 0.167 |
| R248W | 0.900 | 0.500 | 0.400 |
| T211I | 0.300 | 0.250 | 0.167 |
| Y220C | 0.500 | 0.250 | 0.167 |
| Y220D | 0.500 | 0.250 | 0.167 |

# Supplementary Table 5

Supplementary Table 5 can be downloaded at https://zenodo.org/record/8412277.

# Supplementary Table 6

| The effect of background ensemble size | | | |
| --- | --- | --- | --- |
|  |  |  |  |
|  | Size of background distribution data | AUC of ROC | AUC of PR |
| Te-S1 | 1000 | 0.842 | 0.879 |
|  | 2000 | 0.842 | 0.878 |
|  | 5000 | 0.842 | 0.879 |
|  | 10000 | 0.842 | 0.878 |
|  |  |  |  |
| Te-S2 | 1000 | 0.896 | 0.892 |
|  | 2000 | 0.896 | 0.890 |
|  | 5000 | 0.896 | 0.890 |
|  | 10000 | 0.897 | 0.890 |
|  |  |  |  |
| Te-S3 | 1000 | 0.805 | 0.850 |
|  | 2000 | 0.806 | 0.851 |
|  | 5000 | 0.806 | 0.852 |
|  | 10000 | 0.806 | 0.853 |
|  |  |  |  |
| Te-S4 | 1000 | 0.926 | 0.937 |
|  | 2000 | 0.926 | 0.938 |
|  | 5000 | 0.926 | 0.937 |
|  | 10000 | 0.926 | 0.937 |

# Supplementary Table 7

| Testing pMHC-BERT as an epitope-MHC-I binding predictor | | | | |
| --- | --- | --- | --- | --- |
|  |  |  |  |  |
|  | AUC of ROC * | p-value | PPV 1% * | p-value |
| mhcflurry2.0 vs. pMHC-BERT | 28 vs. 48 | 0.006 | 23 vs. 53 | 0.001 |
| MixMHCpred2.2.rank vs. pMHC-BERT | 29 vs. 47 | 0.001 | 58 vs. 18 | < 0.001 |
| NetMHCpan4.1.EL vs. pMHC-BERT | 24 vs. 52 | < 0.001 | 51 vs. 25 | 0.002 |
| NetMHCpan4.1.BA vs. pMHC-BERT | 4 vs. 72 | < 0.001 | 10 vs. 66 | < 0.001 |
|  |  |  |  |  |
| * In this two-column comparison, the second number represents the count of cases where pMHC-BERT exhibited superior performance to the competitor algorithm among the 76 testing samples. For instance, in B4, pMHC-BERT outperformed MHCflurry2.0 in 48 out of 76 test samples from the Te-pMHC dataset, as measured by the AUC-ROC performance metric. | | | | |
| p-value P-values were determined using the Wilcoxon signed-rank test. | | | | |

# Supplementary Table 8A

| The influence of the hyper-parameters (TCR-BERT, pMHC-BERT) | | | | | | |  |
| --- | --- | --- | --- | --- | --- | --- | --- |
|  |  |  |  |  |  |  |  |
|  | lr | batchsize | head | layer | embedding | AUC of ROC | AUC of PR |
| Te-S1 | 0.00005 | 512 | 8 | 4 | 256 | 0.842 | 0.879 |
|  | 0.00001 | 512 | 8 | 4 | 256 | 0.841 | 0.875 |
|  | 0.0001 | 512 | 8 | 4 | 256 | 0.831 | 0.853 |
|  | 0.0005 | 512 | 8 | 4 | 256 | 0.490 | 0.493 |
|  | 0.00005 | 256 | 8 | 4 | 256 | 0.837 | 0.869 |
|  | 0.00005 | 1024 | 8 | 4 | 256 | 0.834 | 0.864 |
|  | 0.00005 | 512 | 4 | 4 | 256 | 0.823 | 0.844 |
|  | 0.00005 | 512 | 6 | 4 | 256 | 0.823 | 0.849 |
|  | 0.00005 | 512 | 10 | 4 | 256 | 0.831 | 0.864 |
|  | 0.00005 | 512 | 12 | 4 | 256 | 0.821 | 0.835 |
|  | 0.00005 | 512 | 8 | 2 | 256 | 0.831 | 0.863 |
|  | 0.00005 | 512 | 8 | 6 | 256 | 0.829 | 0.862 |
|  | 0.00005 | 512 | 8 | 4 | 128 | 0.832 | 0.861 |
|  | 0.00005 | 512 | 8 | 4 | 512 | 0.826 | 0.828 |
|  |  |  |  |  |  |  |  |
| Te-S2 | 0.00005 | 512 | 8 | 4 | 256 | 0.896 | 0.892 |
|  | 0.00001 | 512 | 8 | 4 | 256 | 0.913 | 0.912 |
|  | 0.0001 | 512 | 8 | 4 | 256 | 0.874 | 0.852 |
|  | 0.0005 | 512 | 8 | 4 | 256 | 0.483 | 0.494 |
|  | 0.00005 | 256 | 8 | 4 | 256 | 0.907 | 0.895 |
|  | 0.00005 | 1024 | 8 | 4 | 256 | 0.925 | 0.916 |
|  | 0.00005 | 512 | 4 | 4 | 256 | 0.870 | 0.850 |
|  | 0.00005 | 512 | 6 | 4 | 256 | 0.902 | 0.901 |
|  | 0.00005 | 512 | 10 | 4 | 256 | 0.908 | 0.885 |
|  | 0.00005 | 512 | 12 | 4 | 256 | 0.907 | 0.891 |
|  | 0.00005 | 512 | 8 | 2 | 256 | 0.876 | 0.859 |
|  | 0.00005 | 512 | 8 | 6 | 256 | 0.911 | 0.907 |
|  | 0.00005 | 512 | 8 | 4 | 128 | 0.866 | 0.871 |
|  | 0.00005 | 512 | 8 | 4 | 512 | 0.886 | 0.867 |

| Te-S3 | 0.00005 | 512 | 8 | 4 | 256 | 0.805 | 0.850 |
| --- | --- | --- | --- | --- | --- | --- | --- |
|  | 0.00001 | 512 | 8 | 4 | 256 | 0.816 | 0.846 |
|  | 0.0001 | 512 | 8 | 4 | 256 | 0.817 | 0.846 |
|  | 0.0005 | 512 | 8 | 4 | 256 | 0.537 | 0.529 |
|  | 0.00005 | 256 | 8 | 4 | 256 | 0.827 | 0.846 |
|  | 0.00005 | 1024 | 8 | 4 | 256 | 0.825 | 0.853 |
|  | 0.00005 | 512 | 4 | 4 | 256 | 0.783 | 0.835 |
|  | 0.00005 | 512 | 6 | 4 | 256 | 0.782 | 0.796 |
|  | 0.00005 | 512 | 10 | 4 | 256 | 0.808 | 0.833 |
|  | 0.00005 | 512 | 12 | 4 | 256 | 0.833 | 0.849 |
|  | 0.00005 | 512 | 8 | 2 | 256 | 0.832 | 0.857 |
|  | 0.00005 | 512 | 8 | 6 | 256 | 0.833 | 0.855 |
|  | 0.00005 | 512 | 8 | 4 | 128 | 0.788 | 0.830 |
|  | 0.00005 | 512 | 8 | 4 | 512 | 0.795 | 0.763 |
|  |  |  |  |  |  |  |  |
| Te-S4 | 0.00005 | 512 | 8 | 4 | 256 | 0.926 | 0.937 |
|  | 0.00001 | 512 | 8 | 4 | 256 | 0.936 | 0.950 |
|  | 0.0001 | 512 | 8 | 4 | 256 | 0.889 | 0.878 |
|  | 0.0005 | 512 | 8 | 4 | 256 | 0.474 | 0.487 |
|  | 0.00005 | 256 | 8 | 4 | 256 | 0.932 | 0.948 |
|  | 0.00005 | 1024 | 8 | 4 | 256 | 0.929 | 0.947 |
|  | 0.00005 | 512 | 4 | 4 | 256 | 0.897 | 0.880 |
|  | 0.00005 | 512 | 6 | 4 | 256 | 0.911 | 0.923 |
|  | 0.00005 | 512 | 10 | 4 | 256 | 0.912 | 0.922 |
|  | 0.00005 | 512 | 12 | 4 | 256 | 0.922 | 0.935 |
|  | 0.00005 | 512 | 8 | 2 | 256 | 0.902 | 0.907 |
|  | 0.00005 | 512 | 8 | 6 | 256 | 0.931 | 0.943 |
|  | 0.00005 | 512 | 8 | 4 | 128 | 0.838 | 0.879 |
|  | 0.00005 | 512 | 8 | 4 | 512 | 0.861 | 0.813 |
| * The first row in each sub-table represents the selected parameters in the current model | | | | | | |  |

# Supplementary Table 8B

| The influence of the hyper-parameters (TCR-pMHC prediction model) | | | | | |
| --- | --- | --- | --- | --- | --- |
|  |  |  |  |  |  |
|  | lr | batchsize | model | AUC of ROC | AUC of PR |
| Te-S1 | 0.0005 | 256 | base | 0.842 | 0.879 |
|  | 0.0001 | 256 | base | 0.838 | 0.877 |
|  | 0.00001 | 256 | base | 0.834 | 0.873 |
|  | 0.0005 | 512 | base | 0.836 | 0.872 |
|  | 0.0005 | 128 | base | 0.839 | 0.872 |
|  | 0.0005 | 256 | Larger MLP | 0.810 | 0.784 |
|  | 0.0005 | 256 | Smaller MLP | 0.846 | 0.879 |
|  | 0.0005 | 256 | Embedding dimensional mapping | 0.841 | 0.872 |
|  | 0.0005 | 256 | Length mapping | 0.826 | 0.866 |
|  | 0.0005 | 256 | Larger mapping | 0.833 | 0.843 |
|  |  |  |  |  |  |
| Te-S2 | 0.0005 | 256 | base | 0.896 | 0.892 |
|  | 0.0001 | 256 | base | 0.856 | 0.827 |
|  | 0.00001 | 256 | base | 0.880 | 0.864 |
|  | 0.0005 | 512 | base | 0.887 | 0.874 |
|  | 0.0005 | 128 | base | 0.884 | 0.862 |
|  | 0.0005 | 256 | Larger MLP | 0.866 | 0.819 |
|  | 0.0005 | 256 | Smaller MLP | 0.899 | 0.875 |
|  | 0.0005 | 256 | Embedding dimensional mapping | 0.850 | 0.835 |
|  | 0.0005 | 256 | Length mapping | 0.805 | 0.790 |
|  | 0.0005 | 256 | Larger mapping | 0.876 | 0.860 |

| Te-S3 | 0.0005 | 256 | base | 0.805 | 0.850 |
| --- | --- | --- | --- | --- | --- |
|  | 0.0001 | 256 | base | 0.809 | 0.848 |
|  | 0.00001 | 256 | base | 0.793 | 0.840 |
|  | 0.0005 | 512 | base | 0.815 | 0.845 |
|  | 0.0005 | 128 | base | 0.827 | 0.861 |
|  | 0.0005 | 256 | Larger MLP | 0.785 | 0.714 |
|  | 0.0005 | 256 | Smaller MLP | 0.827 | 0.861 |
|  | 0.0005 | 256 | Embedding dimensional mapping | 0.814 | 0.842 |
|  | 0.0005 | 256 | Length mapping | 0.782 | 0.829 |
|  | 0.0005 | 256 | Larger mapping | 0.817 | 0.804 |
|  |  |  |  |  |  |
| Te-S4 | 0.0005 | 256 | base | 0.926 | 0.937 |
|  | 0.0001 | 256 | base | 0.904 | 0.905 |
|  | 0.00001 | 256 | base | 0.924 | 0.929 |
|  | 0.0005 | 512 | base | 0.916 | 0.918 |
|  | 0.0005 | 128 | base | 0.887 | 0.896 |
|  | 0.0005 | 256 | Larger MLP | 0.886 | 0.871 |
|  | 0.0005 | 256 | Smaller MLP | 0.923 | 0.921 |
|  | 0.0005 | 256 | Embedding dimensional mapping | 0.868 | 0.874 |
|  | 0.0005 | 256 | Length mapping | 0.824 | 0.830 |
|  | 0.0005 | 256 | Larger mapping | 0.845 | 0.835 |
| * The first row in each sub-table represents the selected parameters in the current model | | | | |  |

# Supplementary Table 9A

| Detailed Description of Tr-TCR-pMHC and benchmark test sets (TCR Te-S1-4) | | | | |
| --- | --- | --- | --- | --- |
|  |  |  |  |  |
|  | cdr3_length | proportion | AUC of ROC | AUC of PR |
| Te-S1 | All | 100.00% | 0.842 | 0.879 |
|  | 7, 9, 10, 11, 12 | 15.85% | 0.928 | 0.983 |
|  | 13, 14, 15, 16 | 68.86% | 0.821 | 0.849 |
|  | 17, 18, 19, 20, 21 | 15.29% | 0.694 | 0.614 |
|  |  |  |  |  |
| Te-S2 | All | 100.00% | 0.896 | 0.892 |
|  | 9, 10, 11, 12 | 23.95% | 0.922 | 0.923 |
|  | 13, 14, 15, 16 | 74.43% | 0.890 | 0.871 |
|  | 17, 18, 21, 22 | 1.62% |  | 1.000 |
|  |  |  |  |  |
| Te-S3 | All | 100.00% | 0.805 | 0.850 |
|  | 8, 9, 10, 11, 12 | 19.14% | 0.953 | 0.931 |
|  | 13, 14, 15, 16 | 73.17% | 0.795 | 0.827 |
|  | 17, 18, 19, 20 | 7.68% |  | 1.000 |
|  |  |  |  |  |
| Te-S4 | All | 100.00% | 0.926 | 0.937 |
|  | 10, 11, 12 | 17.90% | 0.980 | 0.997 |
|  | 13, 14, 15, 16 | 70.41% | 0.912 | 0.915 |
|  | 17, 18, 19 | 11.69% | 0.675 | 0.212 |

# Supplementary Table 9B

| Detailed Description of Tr-TCR-pMHC and benchmark test sets (epitope Te-S1-4) | | | | | |
| --- | --- | --- | --- | --- | --- |
|  |  |  |  |  |  |
|  | epitipe length | number of case | proportion | AUC of ROC | AUC of PR |
| Te-S1 | All | 2826 | 100.00% | 0.842 | 0.879 |
|  | 8 | 78 | 2.76% | 0.787 | 0.824 |
|  | 9 | 2168 | 76.72% | 0.831 | 0.871 |
|  | 10 | 386 | 13.66% | 0.884 | 0.907 |
|  | 11 | 132 | 4.67% | 0.893 | 0.929 |
|  | 12 | 12 | 0.42% | 0.805 | 0.882 |
|  | 13 | 45 | 1.59% | 0.971 | 0.980 |
|  |  |  |  |  |  |
| Te-S2 | All | 1236 | 100.00% | 0.896 | 0.892 |
|  | 8 | 46 | 3.72% | 0.776 | 0.807 |
|  | 9 | 650 | 52.59% | 0.903 | 0.894 |
|  | 10 | 252 | 20.39% | 0.860 | 0.878 |
|  | 11 | 108 | 8.74% | 0.977 | 0.981 |
|  | 13 | 180 | 14.56% | 0.907 | 0.799 |
|  |  |  |  |  |  |
| Te-S3 | All | 794 | 100.00% | 0.805 | 0.850 |
|  | 8 | 32 | 4.03% | 0.662 | 0.746 |
|  | 9 | 440 | 55.42% | 0.808 | 0.855 |
|  | 10 | 188 | 23.68% | 0.796 | 0.846 |
|  | 11 | 90 | 11.34% | 0.867 | 0.899 |
|  | 13 | 32 | 4.03% | 0.726 | 0.692 |
|  |  |  |  |  |  |
| Te-S4 | All | 676 | 100.00% | 0.926 | 0.937 |
|  | 9 | 554 | 81.95% | 0.928 | 0.932 |
|  | 10 | 100 | 14.79% | 0.900 | 0.934 |
|  | 11 | 14 | 2.07% | 1 | 1 |

# Supplementary Table 9C

| Detailed Description of Tr-TCR-pMHC and benchmark test sets (MHC Te-S1-4) | | | | | |
| --- | --- | --- | --- | --- | --- |
|  |  |  |  |  |  |
|  | HLA type | number of case | proportion | AUC of ROC | AUC of PR |
| Te-S1 | All | 2826 | 100.00% | 0.842 | 0.879 |
|  | HLA-A | 2318 | 82.02% | 0.833 | 0.872 |
|  | HLA-B | 462 | 16.35% | 0.888 | 0.917 |
|  | HLA-C | 32 | 1.13% | 0.926 | 0.924 |
|  |  |  |  |  |  |
| Te-S2 | All | 1236 | 100.00% | 0.896 | 0.892 |
|  | HLA-A | 732 | 59.22% | 0.894 | 0.888 |
|  | HLA-B | 500 | 40.45% | 0.900 | 0.900 |
|  | HLA-C | 4 | 0.32% | 1.000 | 1.000 |
|  |  |  |  |  |  |
| Te-S3 | All | 794 | 100.00% | 0.805 | 0.850 |
|  | HLA-A | 578 | 72.80% | 0.816 | 0.858 |
|  | HLA-B | 192 | 24.18% | 0.809 | 0.849 |
|  | HLA-C | 20 | 2.52% | 0.495 | 0.571 |
|  |  |  |  |  |  |
| Te-S4 | All | 676 | 100.00% | 0.926 | 0.937 |
|  | HLA-A | 559 | 82.69% | 0.928 | 0.934 |
|  | HLA-B | 106 | 15.68% | 0.940 | 0.955 |
|  | HLA-C | 4 | 0.59% | 0.750 | 0.792 |

# Supplementary Table 10

| The influence of the size of training data (TCR-pMHC prediction model） | | | |
| --- | --- | --- | --- |
|  |  |  |  |
|  | data size | AUC of ROC | AUC of PR |
| Te-S1 | 70423 | 0.842 | 0.879 |
|  | 45000 | 0.830 | 0.868 |
|  | 20000 | 0.830 | 0.871 |
|  | 10000 | 0.829 | 0.870 |
|  |  |  |  |
| Te-S2 | 70423 | 0.896 | 0.892 |
|  | 45000 | 0.876 | 0.864 |
|  | 20000 | 0.871 | 0.853 |
|  | 10000 | 0.858 | 0.838 |
|  |  |  |  |
| Te-S3 | 70423 | 0.805 | 0.850 |
|  | 45000 | 0.811 | 0.842 |
|  | 20000 | 0.803 | 0.844 |
|  | 10000 | 0.795 | 0.841 |
|  |  |  |  |
| Te-S4 | 70423 | 0.926 | 0.937 |
|  | 45000 | 0.896 | 0.904 |
|  | 20000 | 0.907 | 0.912 |
|  | 10000 | 0.907 | 0.911 |

# Supplementary Table 11

| The percentage of data for top 5 hot epitopes in Tr-TCR-pMHC | |
| --- | --- |
|  |  |
|  | Percentage |
| KLGGALQAK | 37.65% |
| YVLDHLIVV | 11.16% |
| GLCTLVAML | 9.75% |
| NLVPMVATV | 6.80% |
| LPRRSGAAGA | 6.08% |
|  |  |
| Total: | 71.44% |

# Supplementary Table 12

| The influence of high-frequency ("hot”) epitope in the training set | | | | |
| --- | --- | --- | --- | --- |
|  |  |  |  |  |
|  |  | Percentage of remained traiing data | AUC of ROC | AUC of PR |
| Te-S1 | All | 100.00% | 0.842 | 0.879 |
|  | train without top 1 hot epitopes | 62.35% | 0.837 | 0.873 |
|  | train without top 5 hot epitopes | 28.60% | 0.842 | 0.880 |
|  |  |  |  |  |
| Te-S2 | All | 100.00% | 0.896 | 0.892 |
|  | train without top 1 hot epitopes | 62.35% | 0.912 | 0.913 |
|  | train without top 5 hot epitopes | 28.60% | 0.893 | 0.885 |
|  |  |  |  |  |
| Te-S3 | All | 100.00% | 0.805 | 0.850 |
|  | train without top 1 hot epitopes | 62.35% | 0.834 | 0.857 |
|  | train without top 5 hot epitopes | 28.60% | 0.828 | 0.861 |
|  |  |  |  |  |
| Te-S4 | All | 100.00% | 0.926 | 0.937 |
|  | train without top 1 hot epitopes | 62.35% | 0.930 | 0.945 |
|  | train without top 5 hot epitopes | 28.60% | 0.929 | 0.936 |

# Supplementary Table 13

| Testing models with unseen TCRs | | | |
| --- | --- | --- | --- |
|  |  |  |  |
|  |  | AUC of ROC | AUC of PR |
| Te-S1 | origin model | 0.842 | 0.879 |
|  | retrain with unseen TCR | 0.829 | 0.871 |
|  |  |  |  |
| Te-S2 | origin model | 0.896 | 0.892 |
|  | retrain with unseen TCR | 0.892 | 0.880 |
|  |  |  |  |
| Te-S3 | origin model | 0.805 | 0.850 |
|  | retrain with unseen TCR | 0.813 | 0.851 |
|  |  |  |  |
| Te-S4 | origin model | 0.926 | 0.937 |
|  | retrain with unseen TCR | 0.926 | 0.935 |

# References：

1. de Andrade KC, Lee EE, Tookmanian EM, et al. The TP53 Database: transition from the International Agency for Research on Cancer to the US National Cancer Institute. Cell Death Differ 2022; 29:1071–1073

2. Gonzalez-Galarza FF, McCabe A, Santos EJM dos, et al. Allele frequency net database (AFND) 2020 update: gold-standard data classification, open access genotype data and new query tools. Nucleic Acids Research 2020; 48:D783–D788
